# HTLV-1 basic leucine zipper factor protects cells from oxidative stress by upregulating expression of Heme Oxygenase I

**DOI:** 10.1371/journal.ppat.1007922

**Published:** 2019-06-28

**Authors:** Amanda W. Rushing, Blake Rushing, Kimson Hoang, Stephanie V. Sanders, Jean-Marie Péloponèse, Nicholas Polakowski, Isabelle Lemasson

**Affiliations:** 1 Department of Microbiology and Immunology, Brody School of Medicine, East Carolina University, Greenville, North Carolina, United States of America; 2 Institut de Recherche en Infectiologie de Montpellier, Centre National de la Recherche Scientifique, Université de Montpellier, Montpellier, France; Imperial College London, UNITED KINGDOM

## Abstract

Adult T-cell Leukemia (ATL) is a lymphoproliferative disease of CD4^+^ T-cells infected with Human T-cell Leukemia Virus type I (HTLV-1). With the exception of allogeneic hematopoietic stem cell transplantation, there are no effective treatments to cure ATL, and ATL cells often acquire resistance to conventional chemotherapeutic agents. Accumulating evidence shows that development and maintenance of ATL requires key contributions from the viral protein, HTLV-1 basic leucine zipper factor (HBZ). In this study we found that HBZ activates expression of Heme Oxygenase 1 (HMOX-1), a component of the oxidative stress response that functions to detoxify free heme. Transcription of *HMOX1* and other antioxidant genes is regulated by the small Mafs. These cellular basic leucine zipper (bZIP) factors control transcription by forming homo- or heterodimers among themselves or with other cellular bZIP factors that then bind Maf responsive elements (MAREs) in promoters or enhancers of antioxidant genes. Our data support a model in which HBZ activates *HMOX1* transcription by forming heterodimers with the small Mafs that bind MAREs located in an upstream enhancer region. Consistent with this model, we found that HMOX-1 is upregulated in HTLV-1-transformed T-cell lines and confers these cells with resistance to heme-induced cytotoxicity. In this context, HBZ-mediated activation of HMOX-1 expression may contribute to resistance of ATL cells to certain chemotherapeutic agents. We also provide evidence that HBZ counteracts oxidative stress caused by two other HTLV-1-encoded proteins, Tax and p13. Tax induces oxidative stress as a byproduct of driving mitotic expansion of infected cells, and p13 is believed to induce oxidative stress to eliminate infected cells that have become transformed. Therefore, in this context, HBZ-mediated activation of HMOX-1 expression may facilitate transformation. Overall, this study characterizes a novel function of HBZ that may support the development and maintenance of ATL.

## Author summary

Human T-cell Leukemia Virus type I (HTLV-1) infection causes a fatal form of leukemia known as Adult T-cell Leukemia (ATL). Given that most current anti-cancer drugs fail to eradicate the leukemic cells in ATL patients, novel treatment strategies are required. One approach is to target functions of one of the proteins produced by the virus, known as HBZ, which is believed to contribute to the development of ATL and to the survival of leukemic cells in ATL patients. In this study, we found that HBZ increases the expression of the cellular enzyme, HMOX-1, which plays a central role in curbing oxidative stress. In the cell, oxidative stress leads to DNA, protein and lipid damage that can ultimately trigger cell-death. HBZ activates HMOX-1 expression at the level of transcription by commandeering a set of cellular transcription factors known as the small Mafs. We provide evidence that increased HMOX-1 levels in ATL cells may counteract effects of anti-cancer drugs that act by inducing oxidative stress. Additionally, we show that high HMOX-1 levels may dissipate oxidative stress caused by other HTLV-1 proteins, thereby promoting the general survival of HTLV-1-infected cells. Given these results, targeting HMOX-1 might be considered as an ATL patient-treatment option.

## Introduction

The accumulation of reactive oxygen (ROS) and nitrogen species (RNS) is known to induce damage to cellular structures, including genetic material. Oxidative DNA damage can result in cell cycle arrest, the induction of replicative senescence, and initiation of apoptosis [1]. To avoid these outcomes, expression of free radical- and metal-scavenging enzymes is induced in response to oxidative stress as a means of limiting cellular damage. The induction of the antioxidant response is largely regulated by the Cap’n’Collar (CNC) transcription factor, NF-E2-related factor 2 (Nrf2), and the small musculoaponeurotic fibrosarcoma (Maf) proteins, MafF, MafG, and MafK. The small Mafs are expressed from different genes; however, they display a high degree of similarity and appear to be functionally redundant [2]. Upon sensing oxidative stress, Nrf2 and small Mafs form heterodimers that activate transcription of antioxidant genes by binding various types of antioxidant response elements (AREs) located in the promoters and/or enhancers of these genes [2, 3]. Though several variations of the ARE have been characterized, all contain a consensus core sequence 5’-TGA[C/G]NNNGC-3’ [2, 3]. Notably, chromatin immunoprecipitation-deep sequencing (ChIP-Seq) analyses of Nrf2 and MafG genomic binding sites that Nrf2/small Maf heterodimers frequently occupy sites containing the sequence 5’-TGCTGA[C/G]TCAGCA-3’, termed Maf recognition elements (MAREs)[4–6].

Though the upregulation of antioxidant enzymes is an important cancer-prevention mechanism in healthy cells, constitutive overexpression of these proteins has been reported in a variety of malignancies. Over-activation of the oxidative stress response in these situations is associated with the development of drug and radiation resistance, increased metastasis, and with poor patient outcomes [7–9]. Heme Oxygenase I (HMOX-1) is one of the antioxidant proteins implicated in mediating these effects. HMOX-1 is a heme-metabolizing enzyme that is a vital component of the iron recycling system [10]. However, HMOX-1 overexpression has been observed in a variety of malignancies, and in these settings, it has been found to promote cancer cell survival and proliferation, and the onset of multi-drug resistance [11–13].

Human T-cell leukemia virus type I (HTLV-1) is a human retrovirus that predominantly infects CD4^+^ T-cells *in vivo*. While most HTLV-1-infected individuals are asymptomatic carriers of the virus, HTLV-1 can cause inflammatory and lymphoproliferative diseases. Tropical spastic paraparesis/HTLV-1-associated myelopathy/ (TSP/HAM) is an insidious neuroinflammatory condition resulting in demyelination of the spinal cord [14]. Though HAM/TSP pathogenesis is not completely understood, evidence suggests that anti-HTLV-1 cytotoxic T-cells (CTLs) target infected lymphocytes that have crossed the blood-brain barrier, resulting in increased pro-inflammatory cytokine production and damage to the surrounding nervous tissue [15]. Adult T-cell leukemia (ATL) is a highly aggressive lymphoproliferative disorder for which there are no effective treatments [16, 17]. Like TSP/HAM, the mechanisms that drive ATL progression are still being elucidated; however, pathogenesis is closely associated with the activities of two, pro-oncogenic viral proteins, Tax and the HTLV-1 basic leucine zipper factor (HBZ)[18].

Tax is known to have roles in regulating proviral gene expression [19], promoting cellular proliferation and replication, and inhibiting apoptosis [18, 20]. Paradoxically, it has also been demonstrated to promote the accumulation of ROS/RNS through the hyperactivation of canonical and non-canonical NF-κB pathways, the upregulation of inducible nitric oxide synthase (iNOS), and through its interaction with ubiquitin-specific protease 10 (USP10) [21–26]. Incidentally, the viral accessory protein p13 has also been linked to increased production of ROS, possibly through its induction of mitochondrial membrane depolarization [22, 27]. Detrimental effects of these two viral proteins is frequently offset by inactivation of the promoter located in the 5’ long-terminal repeat (LTR) of the provirus, which silences expression of all sense strand-encoded proviral genes [28]. However, recent evidence suggests that stressful conditions promote brief reactivation of transcription from the 5’ LTR promoter [29–32]. Therefore, limiting the damage caused by ROS appears to be a lifelong concern of an infected T-cell.

In contrast to all other HTLV-1 proteins, HBZ is expressed from a gene encoded on the antisense strand of the provirus, leading to its constitutive expression throughout HTLV-1 disease progression [33–36]. Certain pro-survival functions of HBZ have been characterized, and its expression is critical for maintaining proliferation of HTLV-1-infected cells [35, 37, 38]. Notably, HBZ has been reported to prevent the induction of apoptosis [39, 40], rescue host cells from NF-κB-induced senescence [41, 42], and promote evasion of Tax-specific CTLs possibly by downregulating sense proviral transcription [43]. These reports confirm that HBZ plays a variety of cytoprotective roles within the host cell, possibly as a means of promoting long-term persistence. However, its involvement in response to Tax and p13-induced oxidative stress is unknown. We questioned whether HBZ modulates the host cell antioxidant response to regulate homeostasis and maintain survival of infected cell clones.

We report that HBZ activates transcription of a set of oxidative stress response genes, among which is *HMOX1*. Focusing on this gene, we found that HBZ-mediated transcriptional activation did not occur through Nrf2/small Maf heterodimers. Instead, our data support a model in which transcriptional activation occurs through the formation of HBZ/small Maf heterodimers that bind MAREs located in an *HMOX1* enhancer region. Consistent with this mechanism, HTLV-1-infected T-cell lines were found to be more resistant to heme cytotoxicity than uninfected T-cell lines, and chemical inhibition of HMOX-1 reduced cell viability of HTLV-1-infected cells. Furthermore, knockdown of HBZ expression increased the oxidative state of HTLV-1-infected T-cells. This observation may be explained by the removal of a safeguard against ROS and RNS accumulation that results from the functions of Tax and p13. Indeed, we found that HTLV-1-infected T-cells lines with active 5’ LTR transcription (expressing Tax and p13) exhibit elevated oxidative states while an infected T-cell line with inactive 5’ LTR transcription does not. Overall, our findings identify a novel HBZ-dependent, pro-survival mechanism that may contribute to HTLV-1 persistence, facilitate successful transformation of infected cells, and help sustain ATL cells in the host.

## Results

### A subset of oxidative stress-induced genes that includes *HMOX1* is upregulated in HBZ-expressing cells

**Fig 1:**
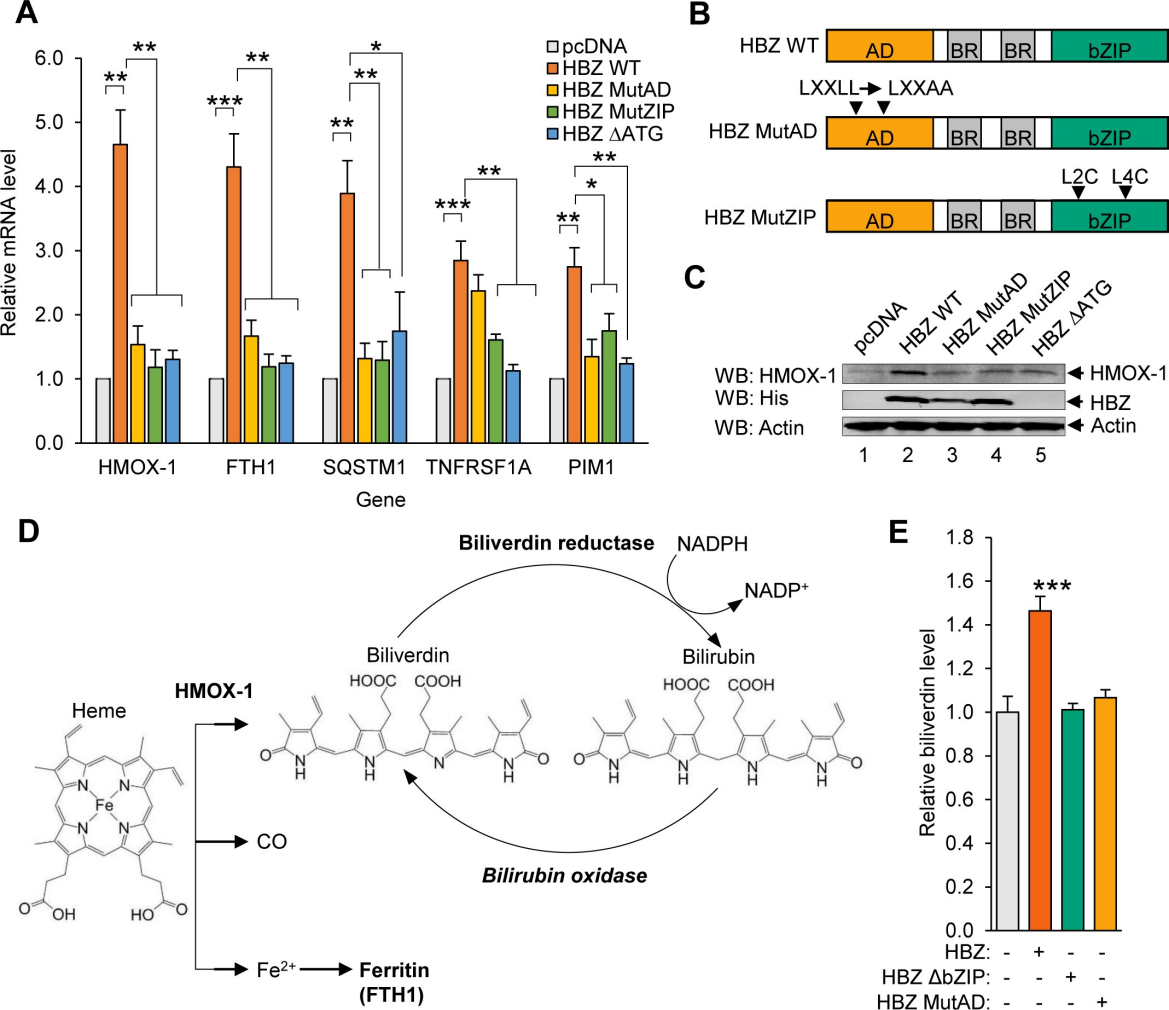
A subset of oxidative stress-induced genes that includes *HMOX1* is upregulated in HBZ-expressing cells. **(A)** A set of oxidative stress-response genes exhibit increased expression in the presence of HBZ. qRT-PCR was used to quantify relative mRNA levels for the indicated genes in HeLa clonal cell lines expressing wild-type HBZ (HBZ WT), an activation domain mutant of HBZ (HBZ MutAD), a leucine zipper domain mutant of HBZ (HBZ MutZIP), or a translational mutant of HBZ (HBZ ΔATG), and in a cell line containing the empty expression vector (pcDNA). *HMOX1* values are averages from three independent experiments; *FTH1*, *SQSTM1*, *TNFRSF1A*, and *PIM1* values are averages from four independent experiments. Data were normalized to the pcDNA sample (set to 1). Error bars represent SEM (two-tailed Student’s t-test, *p≤0.05, **p≤0.01, ***p≤0.001). **(B)** The schematics show the domains of HBZ and the mutants used in this study. Wild type HBZ (HBZ WT) consists of an N-terminal activation domain (AD), centrally located basic regions (BR), and a C-terminal basic leucine zipper domain (bZIP). HBZ MutAD contains LL→AA substitutions in two LXXLL motifs that render the AD defective. HBZ MutZIP contains L→C substitutions in the second and fourth heptad repeats of the ZIP domain, which disrupts dimerization with other bZIP factors. **(C)** HBZ upregulates HMOX-1 expression. Levels of the indicated proteins were evaluated in 30 μg of whole cell extract from each of the HeLa cell lines. The indicated antibodies were used for the Western blot (WB) analysis. **(D)** The schematic shows heme metabolism by HMOX-1. HMOX-1 cleaves the protoporphyrin ring of heme, creating biliverdin, carbon monoxide (CO), and free ferrous iron (Fe^2+^). Ferrous iron is scavenged by Ferritin (FTL/FTH1), and biliverdin reductase converts biliverdin to bilirubin. Bilirubin can be converted back to biliverdin by bilirubin oxidase. **(E)** Higher HMOX-1 levels in HBZ-expressing cells is associated with increased HMOX-1 enzymatic activity in these cells. Biliverdin production was quantified as a measure of HMOX enzyme activity in the indicated HeLa cell lines. Cells were homogenized and lysates were incubated with the HMOX substrate, hemin, and bilirubin oxidase as described in the Materials and Methods. Data are an average of three independent experiments. Error bars represent SEM (two-tailed Student’s t-test, **p≤0.01).

HBZ is known to alter cellular gene expression by affecting the functions of transcriptional regulators; however, an understanding of the downstream physiological impact of these changes remains incomplete. To evaluate HBZ-induced changes in gene expression on a large scale, we previously performed a microarray analysis, comparing gene expression levels between a HeLa clonal cell line that stably expresses HBZ and a cell line containing the empty vector [44]. In manually annotating genes exhibiting potentially higher expression with HBZ, we identified a set of genes that are activated by Nrf2 and small Maf transcription factors under conditions of oxidative stress [3–6]. These genes included *HMOX1* (encodes one of the heme metabolizing enzymes), *FTH1* (encodes the heavy chain of the Ferritin iron transporter), and *SQSTM1* (encodes the stress-induced autophagy receptor, Sequestosome 1). Additionally, we identified *TNFRSF1A* (encodes a tumor necrosis factor α receptor) and *PIM1* (encodes the proto-oncogene serine/threonine-protein kinase), which also appear to be activated by oxidative stress [4–6]. Published ChIP-Seq data support that Nrf2 and MafK bind in proximity to the transcribed regions of these five genes [4–6, 45]. We verified the microarray results using quantitative, reverse-transcriptase PCR (qRT-PCR) to show that the mRNA levels of these oxidative stress-response genes were higher in HeLa cells stably expressing wild type (WT) HBZ than in cells containing the empty vector (pcDNA)(Fig 1A).

HBZ contains central basic regions that facilitate its nuclear import [46] and two transcriptional regulatory domains: an N-terminal activation domain (AD) and a C-terminal basic leucine zipper (bZIP) domain (Fig 1B). The AD interacts with the cellular coactivators p300 and CBP *via* two LXXLL motifs [47, 48]. The leucine zipper (ZIP) of the bZIP domain mediates dimerization with specific cellular bZIP transcription factors [33, 49–52]. To determine which of these domains is important for transcriptional upregulation of the oxidative stress response genes, we performed a qRT-PCR analysis of HeLa clonal cell lines stably expressing HBZ with either LL→AA mutations in both LXXAA motifs of the AD (HBZ MutAD) or L→C mutations in the second and fourth heptad repeats of the ZIP domain (HBZ MutZIP)(Fig 1B). Because the *hbz* mRNA has been reported to affect gene expression [35], we also tested a cell line that does not express the HBZ protein due to an A→T mutation in the ATG start codon of the gene (HBZ ΔATG). We found that expression of all five oxidative stress response genes was significantly reduced in the absence of the HBZ protein (HBZ ΔATG) and by mutations in either the AD or ZIP domain of HBZ (Fig 1A). These results suggest that both transcriptional regulatory domains play roles in upregulating the expression of these genes.

To better understand the mechanism through which oxidative stress response genes are upregulated in HBZ-expressing cells, we focused on *HMOX1*, as transcriptional regulation of this gene and HMOX-1 enzymatic function are well-characterized [10, 53]. This gene was also of interest based on its overexpression in certain cancers and on the association of HMOX-1 activity with survival and proliferation of tumor cells [11–13]. Through Western blot analysis, we first confirmed that the level of HMOX-1 was higher in HBZ-expressing HeLa cells than in cells containing the empty vector (Fig 1C). We then evaluated the enzymatic activity of HMOX-1 using LC-MS to measure the conversion of heme into biliverdin in cell lysates (Fig 1D). In these assays, hemin chloride served as the substrate. In addition, because biliverdin is rapidly converted to bilirubin by biliverdin reductase, bilirubin oxidase was added to facilitate its conversion back into biliverdin. Consistent with the qRT-PCR and Western blot data, cell lysates prepared from HBZ-expressing HeLa cells produced more biliverdin than lysates from cells expressing the HBZ mutants or containing the empty vector (Fig 1E). These results show that elevated HMOX-1 levels in HBZ-expressing cells is accompanied by an increase in HMOX-1 enzyme activity.

### HMOX-1 is upregulated in HTLV-1-infected cells

**Fig 2:**
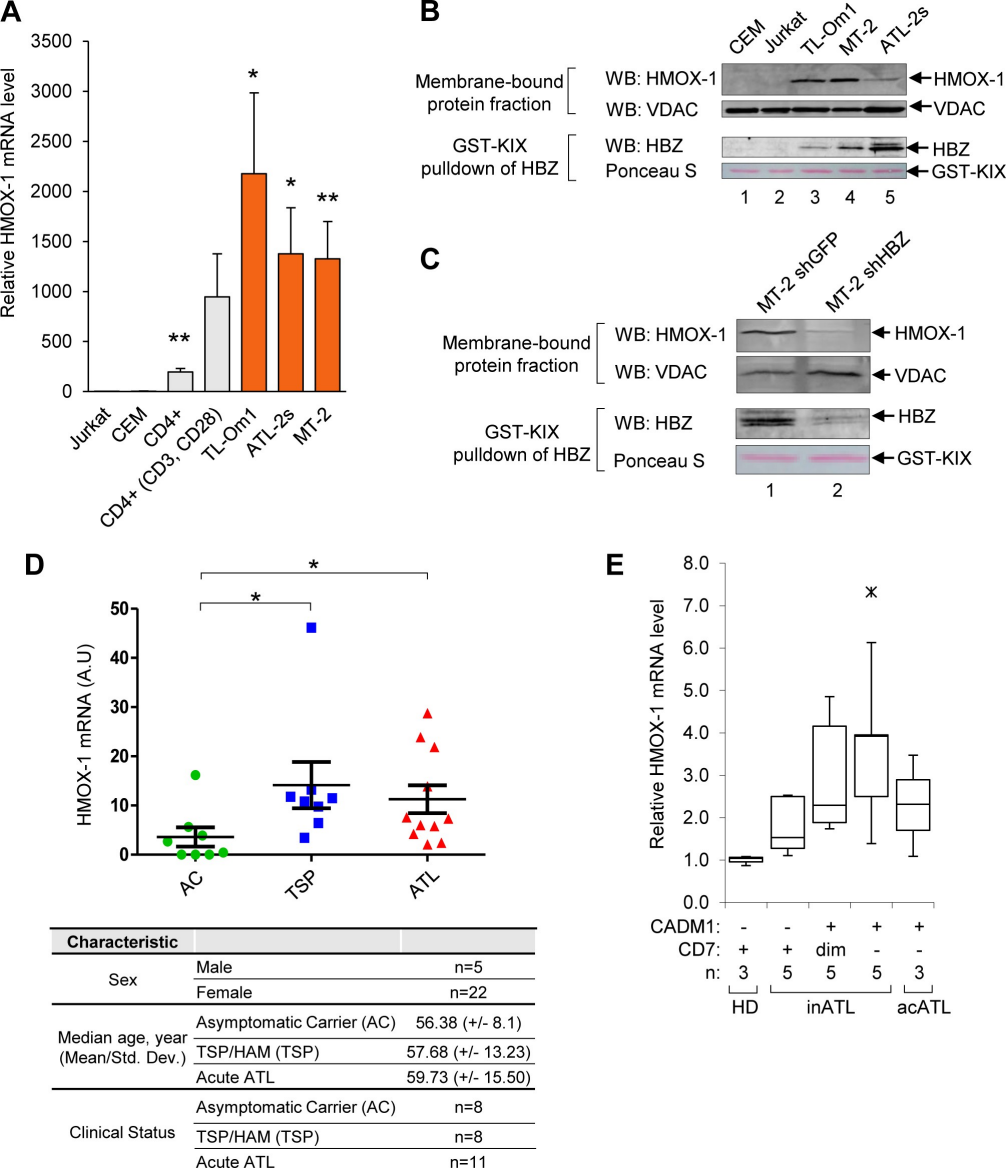
HMOX-1 expression is elevated in HTLV-1-infected T-cell lines and in symptomatic HTLV-1-infected individuals. **(A)** *HMOX1* transcript levels are elevated in HTLV-1-infected T-cells compared to in uninfected T-cells. qRT-PCR was used to quantify relative transcript levels in uninfected T-cell lines (Jurkat and CEM), primary resting and activated CD4^+^ T-cells, and in HTLV-1-infected T-cell lines (TL-Om1, ATL-2s, and MT-2). Data are an average of seven independent experiments, with the exception of primary CD4^+^ data, which are an average of three independent experiments. Error bars represent SEM (two-tailed Student’s t-test, **p≤0.01). **(B)** HMOX-1 protein levels are elevated in HTLV-1-infected T-cells compared to in uninfected T-cells. HMOX-1 and VDAC protein levels were evaluated in 60 μg of the membrane fractions from the indicated T-cell lines using Western blot analysis. HBZ protein levels were determined using recombinant GST-KIX bound to glutathione beads to affinity-purify HBZ from 1 mg of whole cell extracts; eluates were analyzed by Western blot. **(C)** shRNA-mediated knockdown of HBZ reduces HMOX-1 expression. HMOX-1 and VDAC protein levels were evaluated in 60 μg of membrane fractions from MT-2 cells expressing an shRNA targeting HBZ (shHBZ) and from MT-2 cells expressing an shRNA targeting GFP (shGFP). HBZ protein levels were determined using recombinant GST-KIX bound to glutathione beads to affinity-purify HBZ from 300 μg of whole cell extracts; eluates were analyzed by Western blot. The indicated antibodies were used for the Western blot analysis. **(D)** *HMOX1* transcript levels are elevated in the PBMC from HSM/TSP and ATL patients compared to the PBMC from asymptomatic HTLV-1 carriers. qRT-PCR was used to quantify relative *HMOX1* transcript levels in CD8^+^ T-cell-depleted PBMCs isolated from asymptomatic HTLV-1 carriers (AC), TSP/HAM (TSP) patients and acute ATL (ATL) patients. Error bars represent the standard deviation (two-tailed Student’s t-test, *p≤0.05). Subject/patient information is shown below the graph. **(E)** Increasing levels of the *HMOX1* transcript may correlate with ATL disease progression. The graph was generated from published microarray data [60, 61] and shows *HMOX1* transcript levels partitioned by CADM1 and CD7 expression in healthy donors (HD) and patients with acute (acATL) or indolent ATL (inATL). Data obtained using GEOR from the GSE55851 series are represented as a Tukey boxplot where the ends of each whisker are set to 1.5 times the interquartile range above the third quartile and below the first quartile (ж, outlier).

We next used qRT-PCR to measure levels of the *HMOX1* transcript in a panel of uninfected and HTLV-1-infected T-cell lines. The uninfected cells that were tested included the acute T-cell lymphoblastic leukemia cell lines, Jurkat and CEM, as well as resting and anti-CD3/anti-CD28-activated primary CD4^+^ T-cells. The HTLV-1-infected T-cell lines that were tested included MT-2, which was established *in vitro*, and ATL-2s and TL-Om1, which were derived directly from ATL patients. TL-Om1 cells are unique among the HTLV-1-infected cell lines, as the 5’ LTR promoter in these cells is hypermethylated, which blocks expression of sense strand-encoded proteins, including Tax [54]. We compared levels of *HMOX1* transcripts in the primary cells and cell lines to those in Jurkat cells (Fig 2A). Using this approach, we observed a higher level of the transcript in resting, primary CD4^+^ T-cells, which was further elevated in the activated CD4^+^ T-cells. Given that HMOX-1 expression is upregulated during T-cell activation [55], this result was expected. Interestingly, all three HTLV-1-infected T-cell lines, including the TL-Om1 cells that only express HBZ, exhibited transcript levels that were higher than those of the uninfected T-cell lines and the primary T-cells.

Using Western blotting we confirmed that HMOX-1 protein levels are also higher in HTLV-1-infected cells than in uninfected cells (Fig 2B, upper panels). In these assays we analyzed the membrane fractions from the different cell lines, as HMOX-1 is anchored in the membrane of the endoplasmic reticulum [56]. Voltage-dependent anion-selective channel protein (VDAC) was used as the loading control, as it is anchored to the mitochondrial membrane and concentrated in the membrane fraction [57]. We also analyzed HBZ levels in these cell lines using GST-KIX to enrich for HBZ from whole cell extracts (Fig 2B, lower panels)[58].

To evaluate the contribution of HBZ to HMOX-1 expression in HTLV-1-infected cell lines, we compared HMOX-1 protein levels between MT-2 cells expressing an shRNA that targets HBZ and MT-2 cells expressing a control shRNA that targets GFP (Fig 2C, lower panels; S1A Fig)[38, 58]. Analysis of the membrane fractions showed that HMOX-1 protein levels were lower in the HBZ knockdown cells (Fig 2C, upper panels). These results were supported by an *in silico* analysis of microarray data from ATL patient-derived cells (herein denoted as ATL cells) in which HBZ expression was knocked out [59]. In this former study, mRNA levels were quantified seven and eight days following induction of CRISPR/Cas9-mediated editing of *hbz* alleles. Both time points showed a reduction in *HMOX1* mRNA (S1B Fig). Consistent with our findings, these data indicate that reduced HBZ expression is associated with lower HMOX-1 expression.

We next evaluated whether *HMOX1* expression was elevated in PBMCs from patients presenting with an HTLV-1-associated disease. Using qRT-PCR, we compared *HMOX1* transcript levels among CD8^+^ T-cell-depleted PBMCs collected from acute ATL patients (n = 8), HAM/TSP patients (n = 8), and asymptomatic HTLV-1 carriers (n = 11) (Fig 2D). Interestingly, we found *HMOX1* transcript levels were significantly higher in both the ATL and HAM/TSP patient groups compared to asymptomatic carriers. However, we did not observe a significant correlation between *HMOX1* transcript levels and the proviral load in the patient samples (S2 Fig).

To expand upon these patient results, we evaluated *HMOX1* expression from published microarray data sets corresponding to another pool of HTLV-1-infected individuals [60, 61]. In this former study, CD4^+^ T-cells were isolated from healthy donors, asymptomatic HTLV-1 carriers, and from patients with an indolent form of ATL (chronic or smoldering) or patients with acute ATL. By analyzing cell-surface expression of CADM1 and CD7, the authors found that an increase in CADM1 and a decline in CD7 correlate with ATL progression. When we analyzed these data sets, we observed that *HMOX1* transcript levels tend to increase from the CADM1^neg^ CD7^pos^ phenotype to the CADM1^pos^ CD7^neg^ phenotype, suggesting that overexpression of HMOX-1 may be related to ATL disease progression (Fig 2E).

### HBZ does not affect the nuclear localization of Nrf2

**Fig 3:**
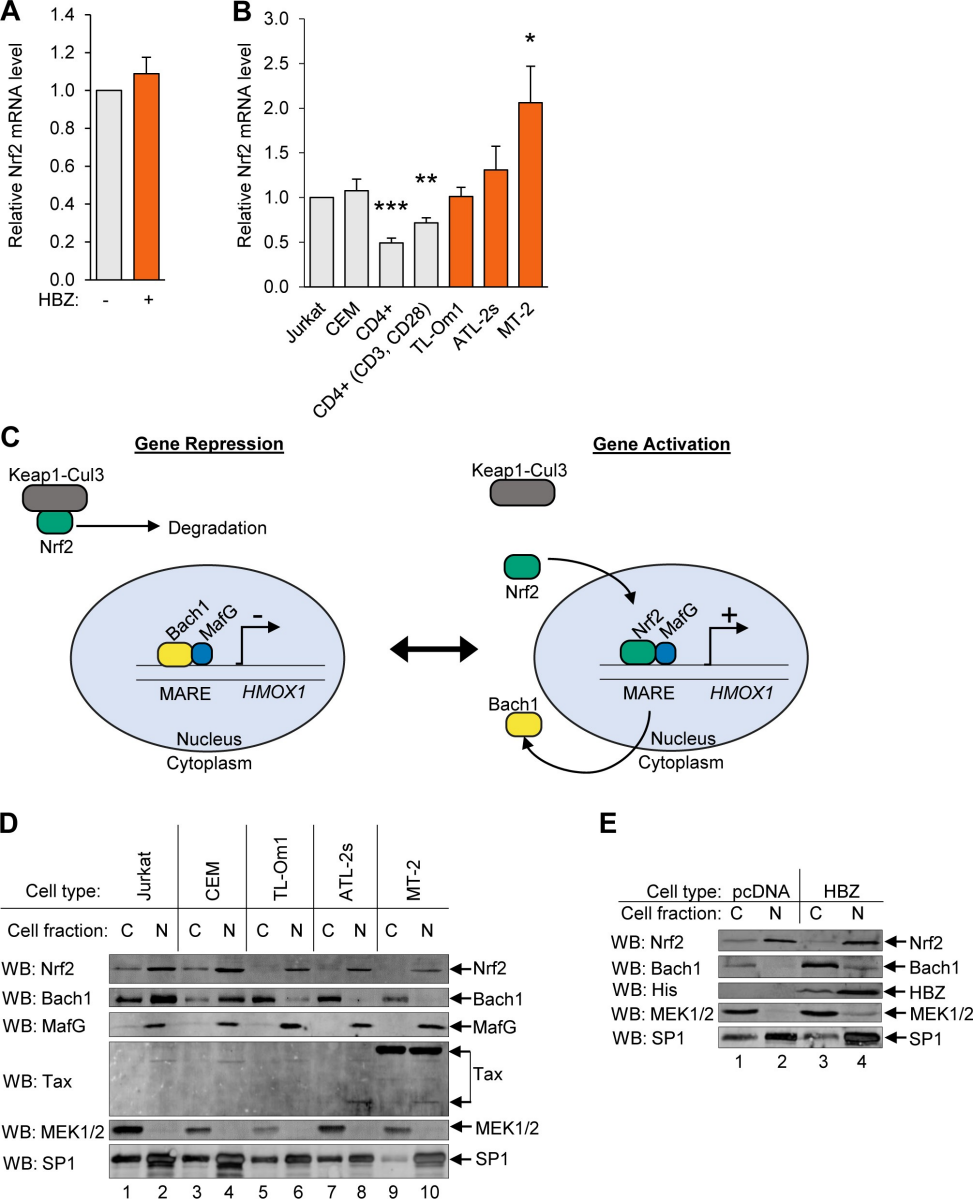
HBZ does not affect the nuclear localization of Nrf2. **(A)** HBZ does not affect *NFE2L2* transcript levels. qRT-PCR was used to quantify relative mRNA levels in the HeLa empty vector (pcDNA) and HBZ-expressing cell lines. Data are an average of four independent experiments and error bars represent SEM (two-tailed Student’s t-test, p≤0.05). **(B)** *NFE2L2* transcript levels are not consistently elevated in HTLV-1-infected T-cells. qRT-PCR was used to quantify relative mRNA levels in uninfected T-cell lines (Jurkat and CEM), resting and activated primary CD4^+^ T-cells, and HTLV-1-infected cell lines (TL-Om1, ATL-2s, and MT-2). Data are an average of five independent experiments, with the exception of primary CD4^+^T-cell data, which are an average of three independent experiments. Error bars represent SEM (two-tailed Student’s t-test, *p≤0.05, **p≤0.01). **(C)** A model of Nfr2 and Bach1 regulation of antioxidant gene expression. In the absence of oxidative stress, Bach1/small Maf heterodimers bound to MARES in antioxidant gene promoters repress transcription. In parallel, the Keap1-Cul3 ubiquitin ligase complex retains Nrf2 in the cytoplasm and induces its rapid degradation. Oxidation releases Nrf2 from Keap1, allowing Nrf2 to accumulate in the nucleus where it replaces Bach1 in the small Maf/MARE complex. Bach1 is thereby exported to the cytoplasm and the newly formed Nrf2/small Maf heterodimers activate transcription. **(D)** Bach1 in HTLV-1-infected T-cells is predominantly cytoplasmic. Levels of the indicated proteins were evaluated in 40 μg of cytoplasmic (C) and nuclear (N) extracts from uninfected T-cell lines (Jurkat and CEM) and HTLV-1-infected T-cell lines (TL-Om1, ATL-2s, and MT-2). The indicated antibodies were used for Western blot analysis. **(E)** HBZ does not affect the cytoplasmic/nuclear distribution of Nrf2. Levels of the indicated proteins were evaluated in 40 μg of cytoplasmic (C) and nuclear (N) extracts from the HeLa empty vector (pcDNA) and HBZ-expressing cell lines. The indicated antibodies were used for Western blot analysis.

Activation of the oxidative stress response is largely dependent on the transcriptional regulator, Nrf2. During oxidative stress, transcription of the *NFE2L2* gene, which encodes Nrf2, increases [3]. In addition, Nrf2, itself, is stabilized and accumulates in the nucleus [3]. We first evaluated whether HBZ-dependent upregulation of antioxidant genes is related to Nrf2 activity by analyzing *NFE2L2* transcript levels in HBZ-expressing cell lines and in HTLV-1-infected T-cell lines. qRT-PCR analysis of *NFE2L2* transcripts in HeLa cells stably expressing HBZ showed levels similar to those found in empty vector control cells (Fig 3A). In addition, among the three HTLV-1-infected cell lines tested, only MT-2 cells exhibited a significantly higher *NFE2L2* transcript level than Jurkat cells (Fig 3B). Therefore, elevated HMOX-1 expression in infected cells and by HBZ alone does not correlate with upregulation of *NFE2L2* gene transcription.

In addition to increasing *NFE2L2* gene transcription, oxidative stress also induces nuclear translocation of Nrf2 [3]. Under homeostatic conditions, Keap1 binds Nrf2 in the cytoplasm and, as an adaptor for a Cul3-dependent ubiquitin ligase complex, mediates proteasomal degradation of Nrf2 (Fig 3C, left schematic). Without Nrf2 in the nucleus, Bach1 dimerizes with MafG (or one of the other two small Mafs) at MARE sites within antioxidant gene promoters, forming a complex that is transcriptionally repressive. During oxidative stress, oxidation of Keap1 disables the Nrf2 degradation process. In parallel, Nrf2 undergoes posttranslational modification that allows it to traffic to the nucleus. These events lead to the accumulation of Nrf2 in the nucleus, and through a separate set of posttranslational modifications, Bach1 is exported from the nucleus (Fig 3C, right schematic). Given that Nrf2 contains an activation domain [62], Nrf2/small Maf dimers bound to MAREs activate antioxidant gene transcription.

We qualitatively compared the cytoplasmic/nuclear distribution of Nrf2, MafG and Bach1 in uninfected and HTLV-1-infected T-cell lines (Fig 3D). In these assays, MEK1/2 served as a marker for the cytoplasmic fraction [62], and SP1 as a marker for the nuclear fraction as it is enriched in this fraction [63, 64]. Surprisingly, we found that the overall levels of Nrf2 were lower in HTLV-1-infected cells, which was more pronounced when comparing the cytoplasmic fractions between the cell sets. These results suggest that Nrf2 undergoes more rapid turnover in infected cells and is, therefore, not mediating the increase in *HMOX1* gene expression in these cells. Similarly, MafG was concentrated in the nucleus in both uninfected and HTLV-1-infected cells, which is consistent with previous reports [65, 66]. Interestingly, when we analyzed the distribution of Bach1, we found that it was highly enriched in the cytoplasmic fractions of the HTLV-1-infected cells, while conversely, it was more prevalent in the nuclear fraction of uninfected cells. These results suggested that, in HTLV-1-infected cells, transcription of antioxidant genes is upregulated through loss of Bach1-mediated repression, surprisingly, without involvement of Nrf2.

Given our evidence that HBZ upregulates *HMOX1* and other oxidative stress-response genes, we tested whether HBZ alone effected a change in the cytoplasmic/nuclear distribution of Bach1 and Nrf2. In comparing HBZ-expressing and empty vector HeLa clones, we did not observe a significant difference in the distribution of Bach1 between the cytoplasm and the nucleus (Fig 3E; S3 Fig). This observation suggests that, in HeLa cells, HBZ alone is unable to further redistribute Bach1 to the cytoplasmic compartment. In addition, HBZ did not affect the cytoplasmic/nuclear distribution of Nrf2 (Fig 3E; S3 Fig). Therefore, HBZ-mediated activation of HMOX-1 expression in these cells may not depend on nuclear localization of Nrf2.

### HBZ interacts with the small Mafs

**Fig 4:**
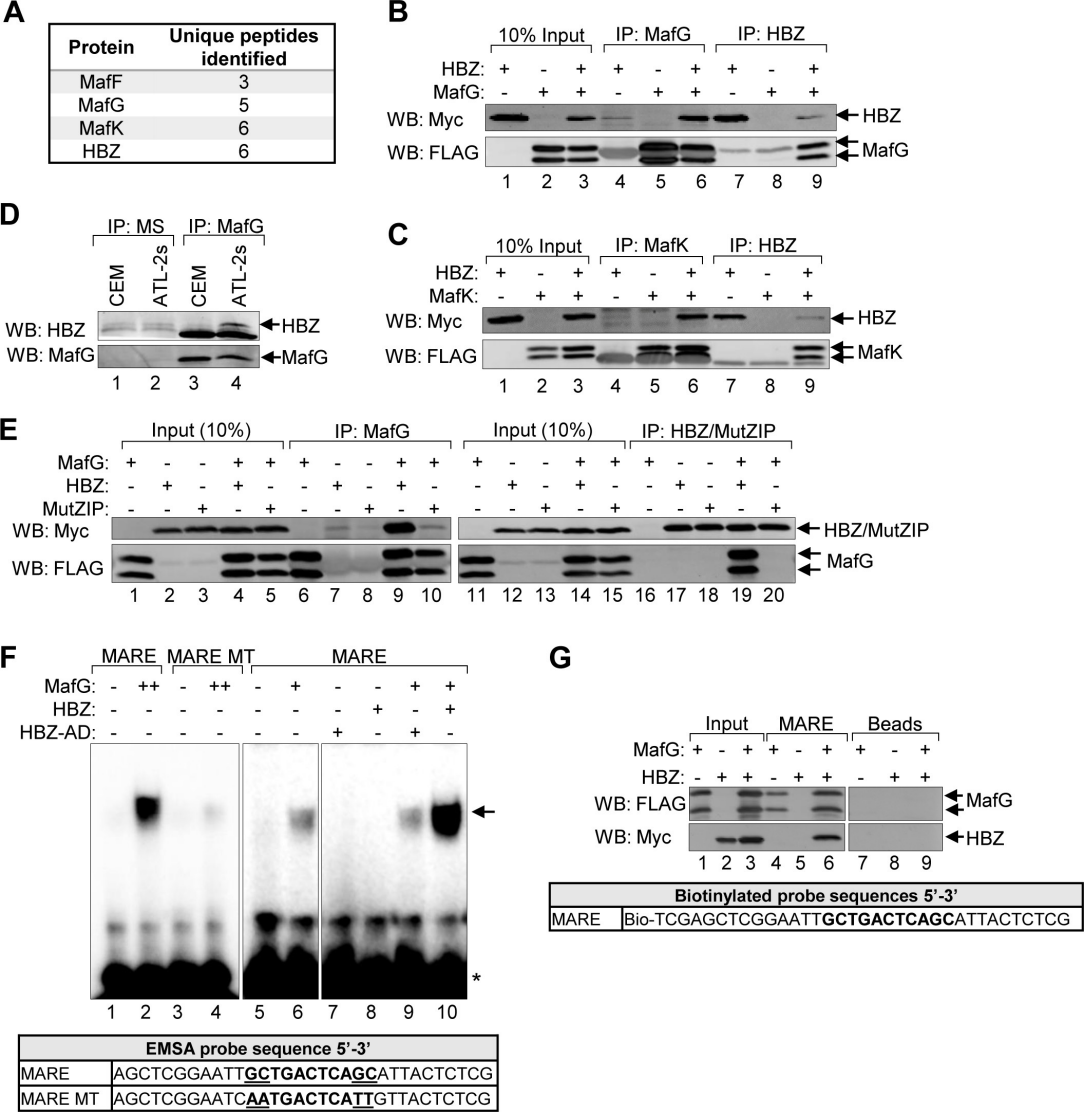
HBZ interacts with the small Mafs to form a DNA-bound complex at MAREs. **(A)** A proteomic analysis identified all three small Mafs (MafF, MafG, and MafK) as potential HBZ-binding partners. A FLAG antibody was used to immunoprecipitate proteins from whole cell extracts prepared from a HeLa clonal cell line expressing HBZ with a C-terminal FLAG tag or a cell line containing the empty vector. Immunoprecipitates were analyzed by LC-MS/MS. Protein identifications were accepted at a false discovery rate of <1%, and a probability of ≥95%, and a maximum of one missed cleavage [68]. The number of unique peptides corresponding to each small Maf identified are shown. Data were obtained from a single experiment. **(B and C)** HBZ interacts with the small Mafs in transfected HEK 293T cells. Cells were transfected with 6 μg pcDNA-HBZ-Myc-His and/or 6 μg pCMV-MafG-FLAG or pCMV-MafK-FLAG (adjusted to 12 μg of total plasmid with the empty vector). HBZ and the small Mafs were immunoprecipitated using anti-Myc (IP: Myc) and anti-FLAG (IP: FLAG) antibodies, respectively. Immunoprecipitates and ten percent of the whole cell extract inputs were analyzed by Western blot using the antibodies indicated. **(D)** Endogenous MafG and HBZ interact in ATL cells. MafG (IP:MafG) was immunoprecipitated from CEM or ATL-2s whole cell extracts. Mouse serum (IP:MS) was used as a negative control for immunoprecipitation. Immunoprecipitates were analyzed by Western blot using the antibodies indicated. **(E)** The small Maf/HBZ interaction requires the ZIP domain of HBZ. HEK 293T cells were transfected with 1 μg pCMV-MafG-FLAG and/or 6 μg pcDNA-HBZ-Myc-His or 6 μg pcDNA-HBZ-MutZIP-Myc-His (adjusted to 12 μg of total plasmid with the empty vector). HBZ/MutZIP and the small Mafs were immunoprecipitated using anti-Myc (IP: Myc) and anti-FLAG (IP: FLAG) antibodies, respectively. Immunoprecipitates and ten percent of the whole cell extract inputs were analyzed by Western blot using the antibodies indicated. **(F)** HBZ augments formation of a MafG/MARE complex *in vitro*. In EMSAs, the indicated combination of recombinant, purified MafG-His (0.5 nM or 1 nM), GST-HBZ (50 nM), and GST-HBZ-AD (50 nM) were combined with a DNA probe containing the consensus T-MARE sequence or one containing AA mutations in the flanking GC boxes (MARE MT; sequences shown below) prior to electrophoretic separation of complexes. The panels shown are from the same gel/same scanned image (identical threshold adjustment). The arrow denotes the shifted protein/DNA complex; the asterisk denotes unbound probe. **(G)** In the presence of MafG, HBZ binds DNA containing the T-MARE sequence. Nuclear extracts prepared from HEK 293T cells transfected with pcDNA-HBZ-Myc-His and/or pCMV-MafG-FLAG were combined with a biotinylated DNA probe containing the T-MARE sequence coupled to streptavidin beads or with streptavidin beads alone (Beads). Bound proteins and 10% of the nuclear extract inputs were analyzed by Western blot using the antibodies indicated.

Because HBZ did not appear to increase HMOX-1 expression through Nrf2, we hypothesized that HBZ regulates antioxidant gene expression through direct interactions with the small Mafs. Indeed, the bZIP domain of HBZ was previously found to interact with MafG *in vitro* [67]. Moreover, we have used a proteomic approach to identify cellular proteins that interact with HBZ, and from this analysis, all three small Mafs arose as potential HBZ-binding partners (Fig 4A)[68]. The small Maf peptides identified are shown in S4 Fig. We confirmed these interactions using co-immunoprecipitation assays in which HEK 293T cells were first transfected with expression vectors for HBZ containing a C-terminal Myc-His tag and either MafG or MafK containing a C-terminal FLAG tag. Using antibodies against the epitope tags, HBZ was co-immunoprecipitated with both small Mafs from whole-cell extracts, and likewise, both small Mafs were co-immunoprecipitated with HBZ (Fig 4B and 4C). The transiently-expressed small Mafs were detected as two bands by Western blot, potentially due to a second in-frame start site within the cDNA sequence. Additionally, we co-immunoprecipitated endogenous HBZ with endogenous MafG from whole-cell lysates prepared from ATL-2s cells (Fig 4D), verifying that the HBZ/small Maf interaction occurs in ATL cells.

As bZIP transcription factors, small Mafs form homo- and heterodimers with other bZIP factors through compatible leucine zipper (ZIP) interactions [69]. To confirm that the ZIP domain of HBZ mediates binding to the small Mafs, we performed co-immunoprecipitation assays from HEK 293T transfected with expression vectors for MafG and wild-type HBZ or HBZ MutZIP. As expected, wild-type HBZ interacted with MafG, but HBZ MutZIP did not (Fig 4E), supporting that the interaction is mediated through the ZIP domains.

### HBZ/small Maf complexes bind the T-MARE sequence *in vitro*

A critical function of the small Mafs is to dimerize with antioxidant response regulators and thereby stably tether these factors to MAREs within antioxidant responsive gene promoters [70]. Many MAREs contain a core TPA-responsive element flanked by GC dinucleotides (GC boxes) as follows: 5’-TGC**TGACTCA**GCA-3’ (the GC boxes are underlined, and the core is in bold)[2, 69]. Given the core sequence, this *cis*-element is denoted as a T-MARE [2]. The GC boxes, in addition to the core element, are critical for small Maf dimers to bind DNA. In the context of small Maf/Nrf2 heterodimers, the 3’ GC box is important for small Maf-binding and 5’ region of the MARE is generally recognized by Nrf2 [2]. Previously, Reinke *et*. *al*. provided biochemical evidence that dimers comprised of the bZIP domains of MafG and HBZ bind the T-MARE sequence [67]. Based on these data, we were interested in testing whether full-length HBZ is incorporated into a small Maf/MARE complex. To assess the DNA-binding activity of a full-length HBZ/small Maf complex, we performed EMSAs using a DNA probe containing the T-MARE sequence flanked by sequences identical to those found in the probes used by Reinke *et*. *al*. (Fig 4F)[67]. As a negative control, we used a DNA probe harboring substitutions in the GC boxes, which negatively impact small Maf-binding [71]. In addition to full-length HBZ, we tested the activation domain of HBZ (HBZ-AD), which does not bind to the small Mafs. Prior to performing EMSAs, we used GST pull-down assays to verify that the recombinant, purified proteins used in these experiments exhibited their characteristic protein binding activities (S5A Fig). As expected, we found that MafG interacts stably with the T-MARE probe but not with the mutant probe (Fig 4F, lanes 2 and 4). In the absence of MafG, both full-length HBZ and HBZ-AD failed to bind the T-MARE probe (Fig 4F, lanes 7–8). However, the combination of full-length HBZ and MafG substantially increased formation of the T-MARE-bound protein complex over that of MafG alone (Fig 4F, lanes 6 and 10). In contrast, HBZ-AD did not alter formation of the MafG/T-MARE complex (Fig 4F, lanes 6 and 9). These data are consistent with previously published observations [67], supporting a mechanism in which HBZ forms heterodimers with the small Mafs that are capable of binding the T-MARE sequence.

To provide support that HBZ is indeed incorporated into the protein complex, we used immobilized DNA-binding assays in which the T-MARE probe was biotinylated and coupled to streptavidin beads that were then incubated with nuclear extracts. The nuclear extracts used were prepared from HEK 293T cells transfected with individual or both expression plasmids for MafG-FLAG and HBZ-Myc-His. As expected, MafG-FLAG bound the MARE probe independently of HBZ (Fig 4G, lane 4). In the absence of MafG-FLAG, we did not detect binding of HBZ to the T-MARE probe (Fig 4G, lane 5), possibly due to competition with Nrf2 and Bach1 for binding to the endogenous small Mafs. However, when MafG-FLAG was also present in extracts, HBZ did bind to the probe (Fig 4G, lane 6). Neither HBZ nor MafG-FLAG bound to DNA-free beads (Fig 4G, lanes 7–9), suggesting that binding to the T-MARE probe was specific. Using the immobilized DNA-binding assay with recombinant, purified proteins produced similar results (S5B Fig). These data further support the mechanism of HBZ-recruitment to T-MAREs through interactions with the small Mafs.

### HBZ is recruited to the *HMOX1* promoter

**Fig 5:**
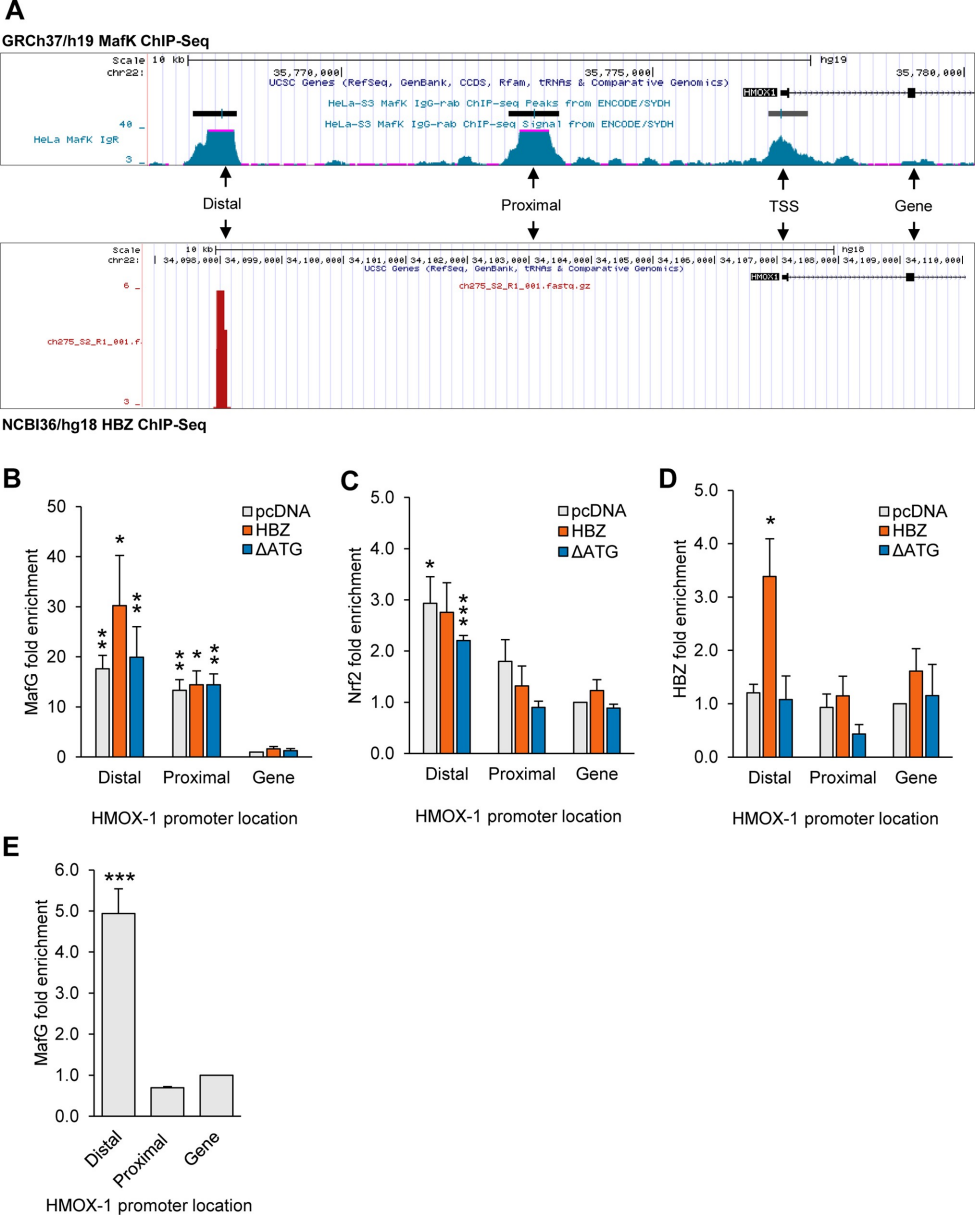
HBZ associates with MAREs in an enhancer upstream of *HMOX1*. **(A)** The *HMOX1* promoter contains two small-Maf-binding regions (Distal and Proximal). The schematic generated from the UCSC genome browser (http://genome.ucsc.edu/, assembly GRCh37/hg19) shows peaks of MafK enrichment within the *HMOX1* promoter in HeLa cells (http://genome.ucsc.edu/; MafK, assembly, GRCh37/hg19). The Distal MafK-binding peak in HeLa cells overlaps with a peak of HBZ enrichment in ATL cells (http://genome.ucsc.edu/; HBZ assembly, NCBI36/hg18 using public data sets GSM2481678 and GSM2481679). Distal and Proximal peak regions and a downstream region used as a ChIP control are shown in the schematic. **(B-D)** HBZ is recruited to the region of the *HMOX1* promoter corresponding to the distal peak of MafK-binding. ChIP assays were performed on chromatin prepared from the indicated HeLa cell lines using antibodies against MafG, Nrf2 and the C-terminal His tag of HBZ. Data are presented as fold enrichment relative to enrichment at the downstream control region in the empty vector (pcDNA) cell line. Data are an average of three independent experiments. Error bars represent SEM (two-tailed Student’s t-test, *p<0.05, **p<0.01, ***p<0.001). **(E)** In TL-Om1 cells, MafG binds to the Distal peak region that is also bound by HBZ. ChIP assays were performed on chromatin prepared from TL-Om1 cells using antibodies against MafG. Data are presented as fold enrichment relative to enrichment at the downstream control region. Data are an average of 5 independent experiments. Error bars represent SEM (two-tailed Student’s t-test, *p<0.05, **p<0.01, ***p<0.001).

Given the important role of the small Mafs in regulating antioxidant gene transcription, we hypothesized that HBZ, through interactions with the small Mafs, directly activated *HMOX1* transcription. ChIP-Seq data available through the UCSC Genome Browser [45] for the MafK revealed two regions of MafK enrichment upstream of *HMOX1*. These regions were originally defined as enhancers [62], and we refer to them as the distal and proximal binding peaks (Fig 5A). Sequence analysis revealed that the distal peak contains three MAREs (Distal 1–3), while the proximal peak contains a single MARE (S6A Fig). To test whether HBZ associates with these peak regions, we performed ChIP assays using HeLa clones that stably express HBZ-Myc-His or the translation-deficient mutant of HBZ (HBZ ΔATG), or contain the empty expression vector (pcDNA). We probed for HBZ using an antibody against its His-epitope tag, and additionally, we probed for MafG and Nrf2 using protein-specific antibodies. qPCR analysis of immunoprecipitated DNA examined the peak regions and a downstream negative control region located within the *HMOX1* gene (Fig 5A). As expected, we found that MafG was highly enriched at both peak regions in all three cell lines (Fig 5B), supporting the concept that the small Mafs may serve as “stand-in” factors that help poise the chromatin for transcription [72]. In contrast to the MafG pattern, significant enrichment of Nrf2 was only observed at the distal peak region with the exception that, in HBZ-expressing cells, Nrf2 was not significantly enriched at this region (Fig 5C). Interestingly, we observed significant enrichment of HBZ at the distal peak region (Fig 5D), suggesting that HBZ increases HMOX-1 expression through association with *HMOX1* gene promoter.

We were also interested in determining whether HBZ binds to the same region of the *HMOX1* promoter in HTLV-1-infected T-cells. Because we do not have a ChIP-grade antibody against HBZ, we analyzed published ChIP-Seq data from experiments designed to identify HBZ-binding sites in the ATL cell line, KK1 [59]. Interestingly, a peak of HBZ-enrichment in the ATL cells overlaps with the distal peak of MafK-enrichment in HeLa cells (Fig 5A). Further analysis of the peak sequences confirmed this observation (S6B Fig) and is consistent with our ChIP results in HeLa cells, providing evidence that HBZ is a direct regulator of *HMOX1* transcription in ATL cells. In conjunction with this analysis we analyzed MafG-binding to the *HMOX1* promoter in TL-Om1 cells. Strikingly, we observed significant enrichment of MafG at the distal peak region where HBZ was found to be enriched according to the *in silico* analysis (Fig 5E). These data suggest that HBZ associates with the *HMOX1* promoter through interactions with the small Mafs.

### HBZ/small Maf complexes bind the distal MAREs in the *HMOX1* promoter

**Fig 6:**
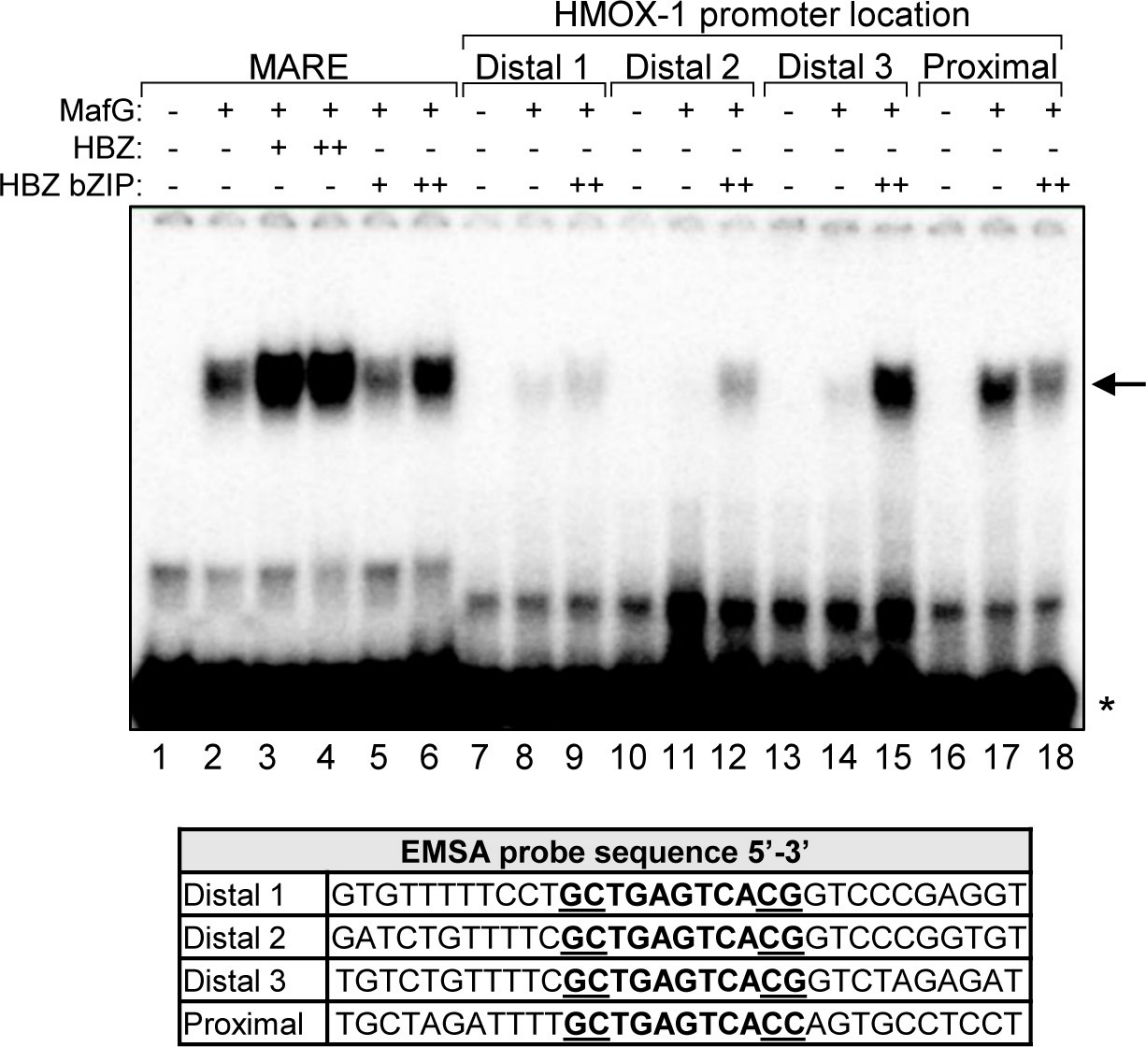
HBZ augments formation of the MafG complex on all three distal MAREs *in vitro*. In EMSAs, the indicated combinations of recombinant, purified MafG-His (0.5 nM), GST-HBZ (50 or 500 nM), and T7-HBZ-bZIP (50 or 500 nM) were combined with the indicated DNA probes (sequences shown below) prior to electrophoretic separation of complexes. The arrow denotes the shifted protein/DNA complex, the asterisk denotes unbound probe.

The distal peak region in the *HMOX1* promoter contains three separate MAREs (Fig 5A). To determine which of these is bound by HBZ/small Mafs heterodimers, we performed EMSAs using probes that encompass each of the three MAREs with 10 or 11 base-pairs of its flanking genomic sequence. These probes were designated Distal 1, Distal 2, and Distal 3 (S6A Fig). In addition, we analyzed binding to a probe encompassing the proximal MARE. To test protein interactions with these probes, we used recombinant, purified MafG and HBZ-bZIP. The bZIP domain of HBZ alone was used in these experiments to further verify that this domain was sufficient for formation of the DNA-bound complex. Importantly, we found that, like the full-length protein, the bZIP domain of HBZ increased formation of the T-MARE/protein complex (Fig 6, lanes 1–6). When we tested binding to the new probes, we found that MafG alone appeared to exhibit a higher affinity for the proximal MARE sequence than for the three distal MARE sequences (Fig 6, lanes 8, 11, 14 and 17). Interestingly, in the case of the three distal MARE probes, HBZ-bZIP increased formation of the protein/DNA complex, an effect that was most pronounced with the Distal 3 probe (Fig 6, lanes 9, 12 and 15). In contrast, HBZ-bZIP diminished formation of a complex with the proximal MARE probe, indicating that HBZ/small Maf heterodimers do not bind the proximal MARE. Overall, these results suggest that HBZ/small Maf heterodimers bind the distal MAREs in the *HMOX1* promoter.

### HBZ activates transcription from T-MAREs

**Fig 7:**
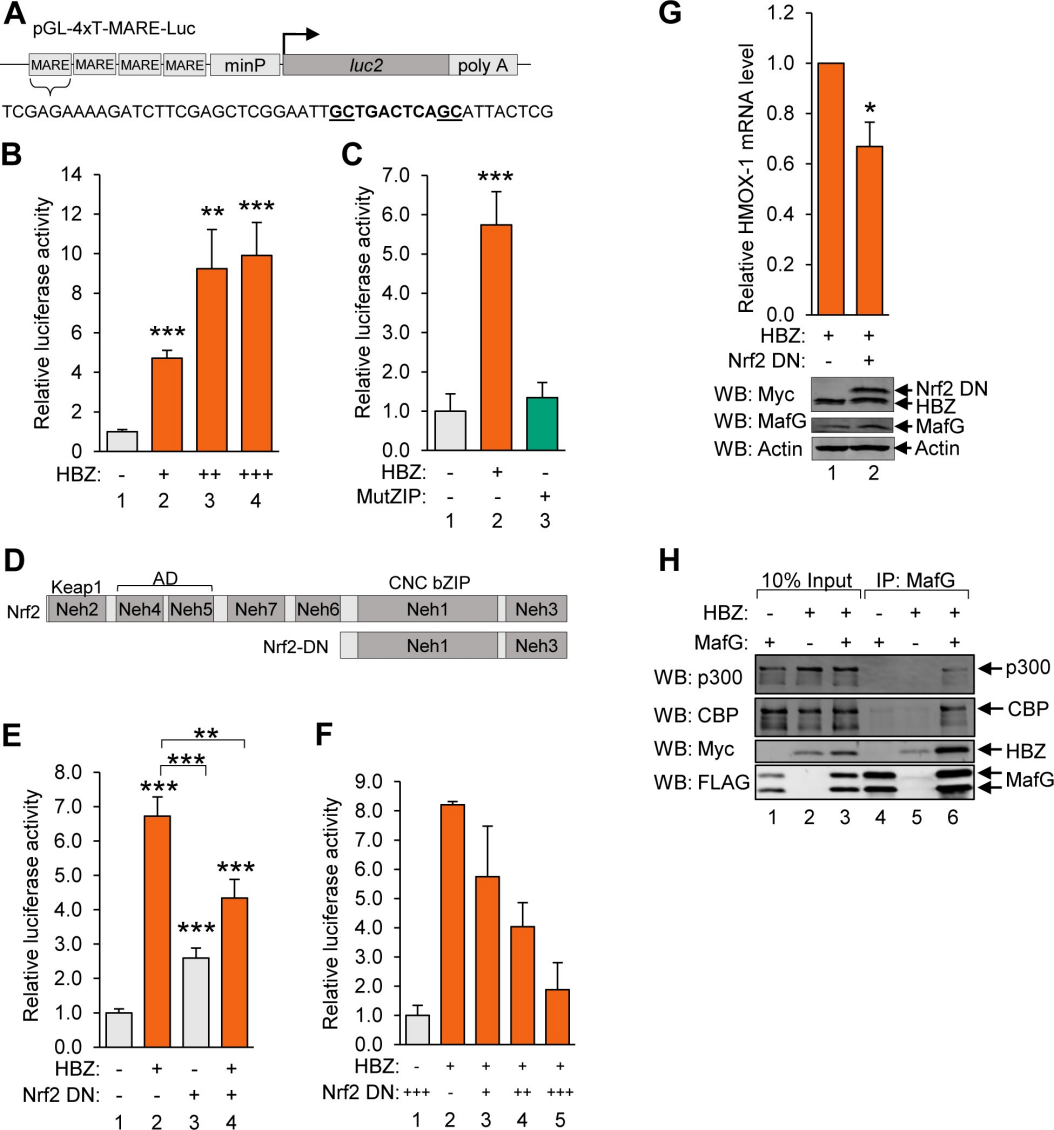
HBZ/small Maf heterodimers activate transcription from T-MAREs. **(A)** The schematic shows the pGL-4xT-MARE-Luc plasmid used in reporter assays. Four tandem repeat sequences containing a T-MARE were cloned into pGL4.26 upstream of the minimal promoter (minP). **(B)** HBZ activates transcription from T-MAREs. Jurkat cells were co-transfected with increasing amounts of pSG-HBZ-Myc and with pGL4.26 or PGL-4xT-MARE-Luc. The graph shows data calculated by subtracting pGL4.26 luminescence values from expression-matched pGL-4xT-MARE-Luc luminescence values. Data are an average of two independent experiments, each of which was performed in triplicate. Error bars represent SEM (two-tailed Student’s t-test, **p≤0.01, ***p≤0.001). **(C)** HBZ activates transcription from T-MAREs, which is dependent on the ZIP domain. Jurkat cells were co-transfected with pSG-HBZ-Myc or pSG-HBZ-MutZIP and pGL4.26 or pGL-4xT-MARE-Luc. The graph shows data calculated by subtracting pGL4.26 luminescence values from expression vector-matched pGL-4xT-MARE-Luc luminescence values. Data are an average of three independent experiments, each of which was performed in triplicate. Error bars represent SEM (two-tailed Student’s t-test, ***p≤0.001). **(D)** The schematic shows full-length Nrf2 with its characterized domains and the region of Nrf2 comprising the dominant negative deletion mutant (Nrf2 DN). The Keap1 binding site, activation domain (AD) and Cap’n’Collar (CNC) bZIP domain encompass Nrf2-ECH homology domain 2 (Neh2), Neh4/5 and Neh1, respectively. **(E)** A dominant negative mutant of Nrf2 suppresses HBZ-mediated transcriptional activation from T-MAREs. Jurkat cells were co-transfected with pSG-HBZ-Myc and/or pcDNA-Nrf2-DN-Myc and pGL4.26 or pGL-4xT-MARE-Luc. The graph shows data calculated by subtracting pGL4.26 luminescence values from expression vector-matched pGL-4xT-MARE-Luc luminescence values. Data are an average of three independent experiments, each of which was in performed in triplicate. Error bars represent SEM (two-tailed Student’s t-test, **p≤0.01, ***p≤0.001). **(F)** Jurkat cells were co-transfected with pSG-HBZ-Myc, increasing amounts of pcDNA-Nrf2-DN-Myc, and pGL4.26 or pGL-4xT-MARE-Luc. The graph shows data calculated by subtracting pGL4.26 luminescence values from expression vector-matched pGL-4xT-MARE-Luc luminescence values. Data shown are representative of three independent experiments, each performed in triplicate. Error bars represent standard deviation. **(G)** A dominant negative mutant of Nrf2 suppresses transcription of endogenous *HMOX1* in Hela cells stably expressing HBZ. HeLa cells stably expressing HBZ-Myc-His were transfected with pMACS CD4 and pcDNA or pcDNA-Nrf2-DN-Myc. Successfully transfected cells were isolated according to ectopic expression of the CD4 surface marker and aliquoted for RNA extraction and, separately, whole cell extract preparation. Relative mRNA levels in control- and Nrf2-DN-transfected cells were quantified by qRT-PCR. Levels of the indicated proteins in 40 μg of whole cell extract were evaluated by Western blot using the antibodies indicated. qRT-PCR data are an average of four independent experiments. Error bars represent SEM (two-tailed Student’s t-test, *p≤0.05). **(H)** HBZ modulates binding of p300/CBP to HBZ/small Maf complexes. HEK 293T cells were transfected with 1 μg pCMV-MafG-FLAG and/or 6 μg pcDNA-HBZ-Myc-His as indicated (adjust to 12 μg with the empty vectors). Proteins were immunoprecipitated using an anti-FLAG antibody to bind MafG. Immunoprecipitates and 10% the whole cell extract inputs were analyzed by Western blot using the antibodies indicated.

To evaluate the transcriptional activity of HBZ from MAREs, we constructed a luciferase reporter plasmid with four tandem repeat sequences containing the T-MARE, which were inserted upstream of a minimal promoter (4xT-MARE; Fig 7A). We then performed reporter assays using Jurkat cells co-transfected with the 4xT-MARE reporter plasmid, or the reporter plasmid containing only the minimal promoter (minP), as well as increasing quantities of an expression vector for wild-type HBZ. Results from these assays showed that HBZ significantly activates transcription from the T-MARES (Fig 7B). In contrast, HBZ MutZIP had no effect on transcription from these sites (Fig 7C). These data show that HBZ activates transcription from T-MAREs, and support a model that it does so through interactions with the small Mafs.

To verify that this transactivation by HBZ depends on the small Mafs, we performed similar luciferase reporter assays with the addition of an Nrf2 dominant negative mutant (Nrf2-DN). This mutant harbors an N-terminal deletion that removes its Keap1-binding site (Neh2) and activation domain (Neh4, Neh5), but retains its CNC-bZIP domain (Fig 7D)[62]. Therefore, Nrf2-DN competes with other small Maf-binding partners, forming transactivation-defective heterodimers with the small Mafs on MAREs. To assess the effects of this mutant on transactivation from the T-MAREs, we co-transfected Jurkat cells with the 4xT-MARE or the minP reporter plasmid and expression vectors for wild-type HBZ and/or Nrf2-DN (Fig 7E). We observed that Nrf2-DN alone slightly increased 4xT-MARE transcription, which may be a de-repressive effect caused by competition between the mutant and Bach1 for small Maf-binding (Fig 7E, lane 3). As expected, HBZ activated 4xT-MARE transcription (Fig 7E, lane 2); however, this effect was significantly diminished in the presence of Nrf2-DN (Fig 7E, lane 4) and was also found to be dose-dependent (Fig 7F). Consistent with these results, ectopic expression of Nrf2-DN in HeLa cells stably expressing HBZ significantly reduced levels of the endogenous *HMOX1* transcript (Fig 7G). These data further support the model that HBZ upregulates transcription from MAREs by forming dimers with the small Mafs.

Though small Mafs are critical regulators of antioxidant gene expression, they lack activation domains. Therefore, gene expression is upregulated by small Mafs when they form MARE-bound heterodimers with Nrf2, which provides these complexes with an activation domain [73, 74]. Given that the activation domain of HBZ functions through interactions with the paralogous coactivators, p300 and CBP [47, 48], we hypothesized that HBZ acts similarly to Nrf2 when bound to the small Mafs. Indeed, as shown in Fig 1A, in comparison to wild-type HBZ, HBZ MutAD exhibited a significant decrease in its ability to activate antioxidant gene expression. Consequently, we tested this hypothesis using co-immunoprecipitation assays to evaluate binding of the coactivators to HBZ/small Maf complexes. HEK 293T cells were transfected with expression vectors for MafG and/or HBZ, and proteins from cell lysates were immunoprecipitated using a MafG antibody. From these assays, we found that p300 and CBP only co-immunoprecipitate with MafG when HBZ is present (Fig 7H), suggesting that HBZ activates antioxidant gene transcription by recruiting p300/CBP to the promoters of these genes.

### HBZ reduces oxidative stress in HTLV-1-infected T-cells

**Fig 8:**
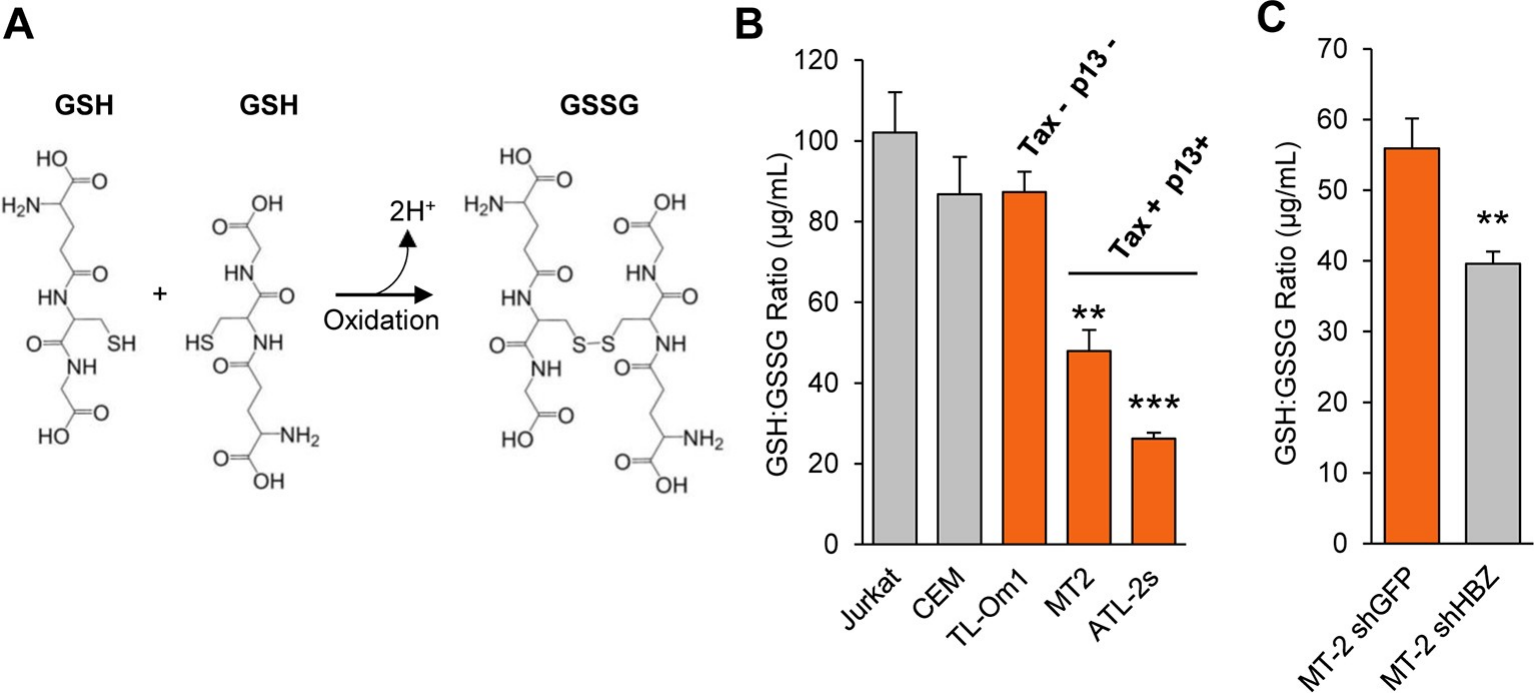
HBZ reduces oxidative stress in HTLV-1-infected T-cells. **(A)** A schematic showing the conversion of reduced glutathione (GSH) to its oxidized form, glutathione disulfide (GSSG) upon exposure to free radicals generated during oxidative stress. **(B)** The oxidative state of HTLV-1-infected T-cells that express Tax and p13 is elevated. Clarified lysates were prepared from uninfected (Jurkat and CEM) and HTLV-1-infected T-cells (TL-Om1, MT-2, and ATL-2s) that had been equalized at 5x10^5^ cells per mL and cultured for 24 hours. Levels of GSH and GSSG in the lysates were quantified by LC-MS and the GSH:GSSG ratios were calculated. Experiments were performed in triplicate and the data shown are an average of two independent experiments. Error bars represent SEM (two-tailed Student’s t-test, **p<0.01, ***p<0.001). **(C)** GSH:GSSG ratios were quantified from MT2 cells expressing an shRNA targeting HBZ (shHBZ) and MT2 cells expressing a control shRNA targeting GFP (shGFP). Experiments were performed in triplicate and the data shown are an average of three independent experiments. Error bars represent SEM (two-tailed Student’s t-test, **p≤0.01).

Previous reports have shown that expression of the HTLV-1-encoded proteins, Tax and p13, results in the accumulation of ROS/RNS [21, 22, 24–27, 75]. Since we found that HBZ upregulates expression of *HMOX1* and other antioxidant genes, we wanted to test whether HBZ protects HTLV-1-infected cells from Tax- or p13-induced oxidation. To examine this possibility, we first measured the ratios of reduced to oxidized glutathione in uninfected and HTLV-1-infected T-cells as a means of assessing the redox states of these cells. Glutathione is a ubiquitous, non-enzymatic antioxidant that, in its reduced form (GSH), is a tripeptide with a single cysteine residue [76]. Upon exposure to free radicals, the thiol group of this cysteine is oxidized, resulting in glutathione disulfide (GSSG) (Fig 8A). In experiments, LC-MS was used to quantify levels of free GSH and GSSG in each cell lines which is reported as a ratio (GSH:GSSG). From this analysis, we found that Jurkat and CEM cells exhibited GSH:GSSG ratios that are within the range reported for normal, unstressed cells (Fig 8B), which for healthy cells, is 100:1 or greater [76–78]. Among the HTLV-1-infected cell lines, ATL-2s and MT-2 cells had significantly lower GSH:GSSG ratios indicative of a more oxidized cellular state. In contrast, TL-Om1 cells, which do not express Tax or p13, exhibited a redox state similar to those of the uninfected cells. This general pattern is consistent with the pro-oxidative effect of the two viral proteins.

We then used the same approach to compare the redox states between MT-2 cells expressing an shRNA that targets HBZ and MT-2 cells expressing an shRNA that targets GFP. We found that the GSH:GSSG ratio was significantly lower in the cells expressing the shRNA that targets HBZ (Fig 8C), indicating that HBZ helps prevent the accumulation of ROS/RNS in HTLV-1-infected cells.

### HBZ-expressing cells display resistance to stress-mediated killing

**Fig 9:**
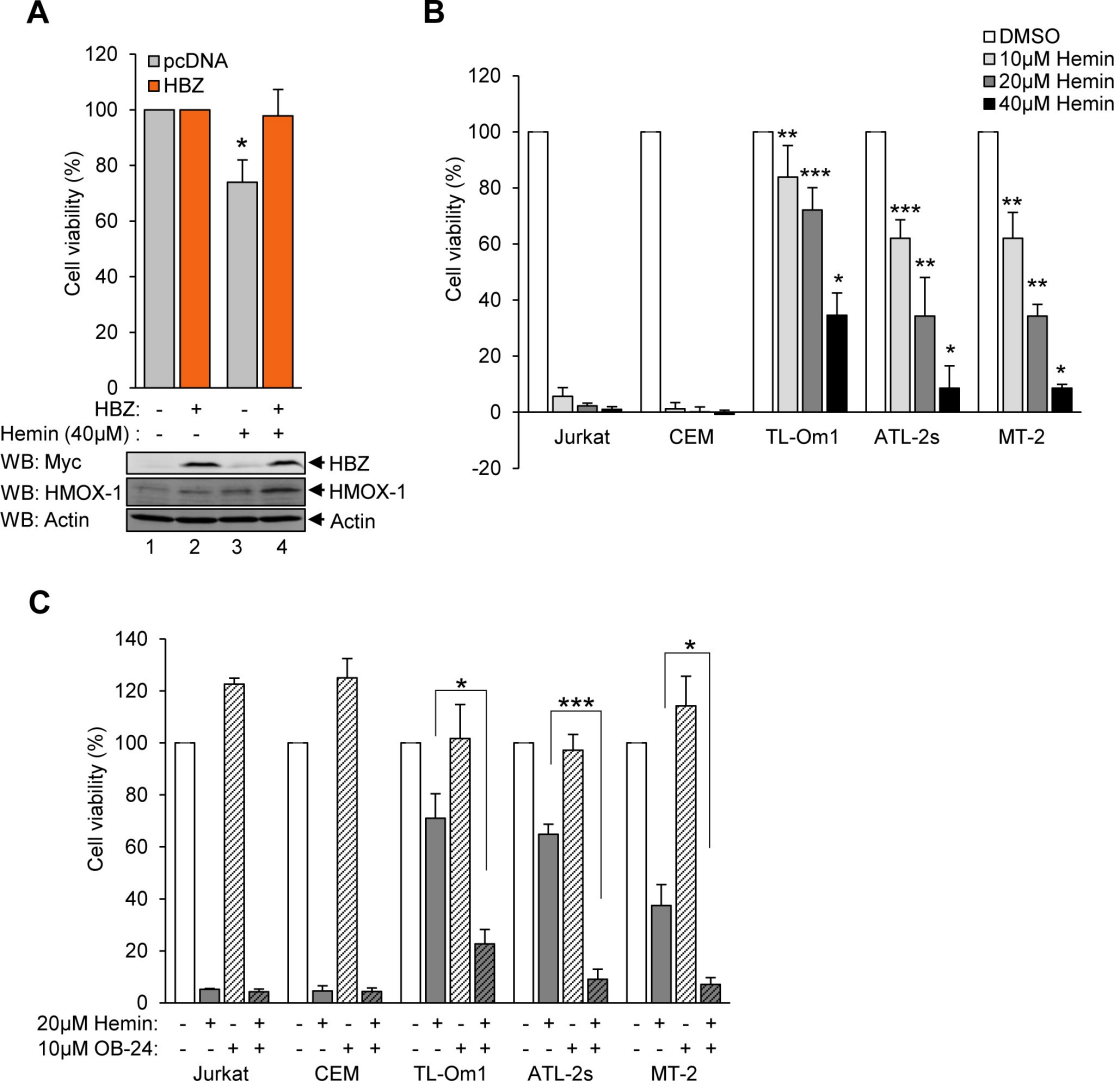
HBZ-expressing cells display resistance to stress-mediated killing. **(A)** HeLa cells expressing HBZ exhibit resistance to the cytotoxic effects of hemin. MTT assays were used to quantify the relative viability of HeLa cells stably expressing HBZ or containing the empty expression vector (pcDNA) cultured in low-serum medium (0.5% serum) with 40 μM hemin or the vehicle control (DMSO). For each experiment, cell-viability was determined from 12 replicates for each treatment of each cell line. Data are the average of three independent experiments and error bars represent SEM (two-tailed Student’s t-test, *p≤0.05). **(B)** HTLV-1-infected T-cells exhibit resistance to the cytotoxic effects of hemin. Relative viability was quantified using alamarBlue on uninfected (Jurkat and CEM) and HTLV-1-infected (TL-Om1, MT-2, and ATL-2s) T-cells cultured in low-serum medium (0.5% serum) with 10, 20 or 40 μM hemin or the vehicle control (DMSO). For each experiment, cell viability was determined from 12 replicates for each treatment of each cell line. Data are the average of three independent experiments and error bars represent SEM (two-tailed Student’s t-test, *p≤0.05, **p≤0.01, ***p≤0.001). **(C)** Inhibition of HMOX-1 sensitizes HTLV-1-infected cells to the cytotoxic effects of hemin. Relative viability was quantified using alamarBlue on uninfected (Jurkat and CEM) and HTLV-1-infected (TL-Om1, MT-2, and ATL-2s) T-cells cultured in low-serum medium (0.5% serum) with the HMOX-1 inhibitor, OB-24 (10 μM), and/or 20 μM hemin. For each experiment, cell-viability was determined from eight replicates for each treatment of each cell line. Data are the average of three independent experiments. Error bars represent SEM (two-tailed Student’s t-test, *p≤0.05, ***p≤0.001).

Constitutive HMOX-1 expression has been demonstrated to confer resistance to cellular stressors, including its substrate heme, as well as to some chemotherapeutic agents [11–13]. Many HTLV-1-infected T-cell lines, including patient-derived ATL cell lines, exhibit increased resistance to a variety of stress-inducing chemotherapy agents, including cisplatin, doxorubicin, and etoposide [79–85]. We hypothesized that the anti-oxidative effect of HBZ is cytoprotective and promotes cell survival during oxidative stress. We used HeLa cells either stably expressing HBZ or carrying the empty vector to test this hypothesis. Cell-viability in each cell line was assessed using MTT assays after challenging cells with hemin to induce iron-mediated oxidative stress. We found that, following hemin-treatment, cells carrying the empty vector exhibited a significant reduction in viability, while HBZ-expressing cells were unaffected by the treatment (Fig 9A).

To expand upon these results, we used alamarBlue cell viability assays to compare the effects of hemin cytotoxicity between uninfected (Jurkat and CEM) and HTLV-1-infected T-cells (TL-Om1, ATL-2s and MT-2). We found that exposure of Jurkat and CEM cells to varying concentrations of hemin cause substantial cell-death, while in comparison, these treatments were significantly less effective at killing the HTLV-1-infected T-cells (Fig 9B).

To test whether the hemin-resistance of the infected cells is dependent on HMOX-1, we used a small molecule inhibitor of HMOX-1, 2-[2-(4-bromophenyl)ethyl]-2-[(1H-imidazol-1-yl)methyl]-1,3-dioxolane hydrochloride (OB-24)[86]. In these experiments, cells were treated with OB-24 and hemin in combination or alone. For uninfected cells, the combination of the two compounds did not decrease cell viability below that produced by hemin alone (Fig 9C). Given our initial findings that these cells are already highly susceptible to hemin-mediated cytotoxicity, this result was expected. In contrast, all three HTLV-1 infected cell lines exhibited a significantly reduction in cell survival with the two compounds together compared to hemin alone (Fig 9C). These results show that the increased expression of HMOX-1 in HTLV-1-infected cells helps avert oxidative stress, and thereby promotes the survival of these cells.

## Discussion

**Fig 10:**
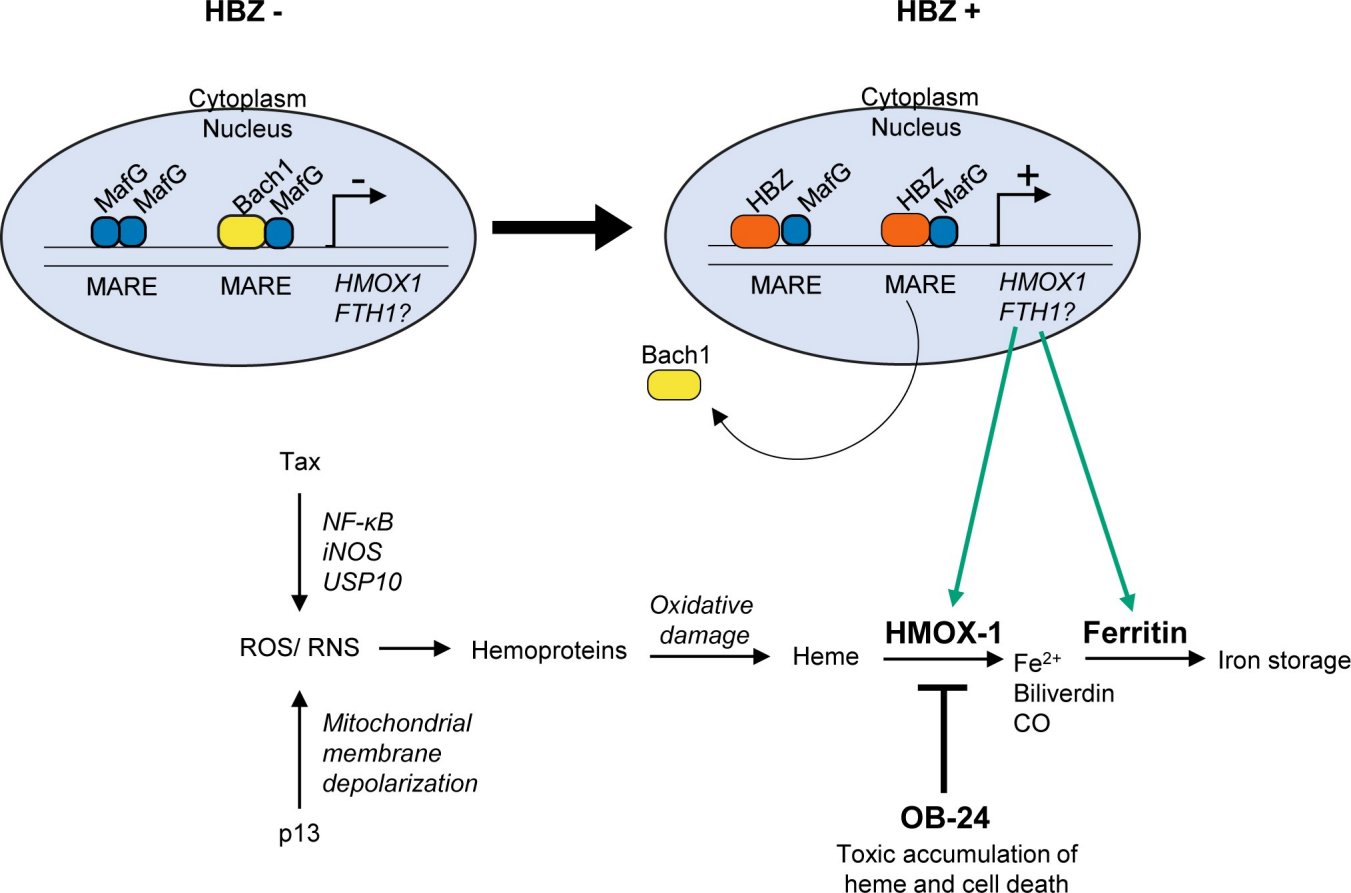
A proposed model for HBZ-mediated HMOX-1 cytoprotection in ATL cells. Oxidative stress, which may originate in part from the activities of virally-encoded Tax and p13 may result in damage to hemoproteins, releasing the heme prosthetic group. The expression of *HMOX1*, and possibly *FTH1*, is upregulated by HBZ/small Maf complexes to promote the detoxification of heme and prevent heme-associated cytotoxicity. The inhibition of HMOX-1 enzymatic activity by the small compound OB-24 promotes cell death, likely through accumulation of heme and the induction of oxidative stress.

In this study, we found that HBZ activates the expression of a group of antioxidant genes that are normally induced by oxidative stress. Using the *HMOX1* gene as a model, we provide evidence that HBZ activates transcription of this gene by forming heterodimers with small Mafs (MafF, MafG or MafK) at MARE sites located in an upstream enhancer. As the small Mafs lack activation domains, they rely on interacting partners, such as Nrf2, to activate transcription [2]. We provide evidence that such a mechanism applies to small Maf/HBZ heterodimers, with HBZ supplying an activation domain that mediates recruitment of the coactivator, p300/CBP, which in turn leads to activation of transcription (Fig 10). This mechanism is remarkable considering that the basic region of the bZIP domain of HBZ lacks consensus amino acid motifs found in other bZIP factors. Given the critical role of the basic region in DNA-binding, heterodimers formed by HBZ and one of a variety of other cellular bZIP factors often fail to bind DNA, and in this context, HBZ functions as a transcriptional repressor [33, 49, 51, 52]. In lieu of this general mechanism, Reinke *et al*. previously provided strong biochemical evidence that heterodimers composed of the bZIP domains of MafG and HBZ exhibit DNA-binding activity [67]. There are multiple ARE subtypes, and in this former study, a T-MARE was used, which consists of a central TRE flanked by GC-boxes [2]. The GC boxes are known to be important for small Maf-binding, and interestingly, according to our results, they may also be important for HBZ-binding. Indeed, our data indicate that HBZ/small Maf heterodimers bind the distal *HMOX1* enhancer AREs (denoted as MAREs here) that contain both 5’ and 3’ GC boxes, but not the proximal ARE that lacks a 3’ GC box. Because substantial sequence variation exists among the AREs, future studies will be needed to clarify which *cis*-acting elements are targeted by HBZ/small Maf heterodimers and, more specifically, the consensus motif recognized by these complexes.

It should be noted that HBZ has also been reported to interact with one of the large Mafs, MafB, which contrary to our observations with small Mafs, inhibits DNA-binding and transcription [87]. While large and small Mafs exhibit high sequence similarity (S4 Fig), large Mafs are distinct in that they contain an activation domain and display tissue-specific expression [88]. In the previous study, the DNA sequence used to assess DNA-binding lacked a 5’ GC box, which may have impaired binding by a MafB/HBZ complex. Interestingly, in the study by Reinke *et al*., heterodimers composed of the bZIP domains of MafB and HBZ did appear to bind the T-MARE sequence, suggesting that HBZ/MafB heterodimers do in fact bind certain AREs [67]. However, the relevance of MafB/HBZ heterodimers will depend on whether MafB is expressed in HTLV-1-infected T-cells, which has not yet been assessed.

In EMSAs HBZ appears to increase the stability of the protein complex formed on ARE sequences. This observation is reminiscent of the effect of the viral protein Tax on CREB in the context of the Tax-responsive element-1 (TxRE1) sites that regulate transcription of the HTLV-1 provirus [19]. In the absence of Tax, CREB binds weakly to these sequences; however, when Tax associates with CREB, TxRE1-bound protein complexes are substantially more stable [89, 90]. This effect of Tax is instrumental to transcriptional activation of the HTLV-1 provirus. In a similar manner stabilization of ARE-bound protein complexes by HBZ may be significant to the mechanism by which HBZ activates transcription from AREs.

Among the antioxidant genes found to be regulated by HBZ, we focused primarily on characterizing downstream effects of increased *HMOX1* expression in relation to HTLV-1 infection and ATL. While HMOX-1 expression is induced by oxidative stress in healthy cells to serve a beneficial and tumor-suppressive role [10], unregulated expression of HMOX-1 acquired by certain cancers is linked to increased cell-survival and proliferation [11–13]. Additionally, HMOX-1 expression has been reported to be induced in response to chemotherapies and radiotherapies, which may help confer cancer cells with multi-drug resistance [11–13]. These adverse effects of HMOX-1 coincide with features of ATL, as ATL patients do not typically respond well to chemotherapeutic regimens, and disease progression and relapse after treatment are often associated with the onset of multi-drug resistance [91]. Moreover, ATL cells and HTLV-1-transformed T-cell lines in culture display resistance to many clinically-relevant anti-cancer drugs [79–85]. In line with these observations, we found that HMOX-1 expression is elevated in HTLV-1-infected T-cell lines and in PBMCs from a set of patients with acute ATL. Furthermore, an analysis of published microarray data [60, 61] suggests that increasing *HMOX1* expression is associated with the worsening of ATL symptoms.

To date, the drug resistance of ATL cells has been associated with viral-mediated activation of pro-survival mechanisms that bypass cell-cycle regulatory checkpoints, inhibit the induction of apoptosis, and increase drug efflux from the cells [79–85]. In this study we identified an additional mechanism that involves resistance to ROS-induced heme cytotoxicity (Fig 10). Heme is released by hemoproteins that are damaged through the effect of ROS. The resulting free heme can then promote peroxidation of membrane lipids, protein fragmentation and DNA damage, ultimately leading to cell death [92]. Through degradation of free heme by HMOX-1, these effects are averted. Indeed, we found that, when challenged with heme, the elevated expression of HMOX-1 in HTLV-1-infected T-cells increased cell-survival. Interestingly, multiple approaches have been used to reduce HMOX-1 expression in different types of cancer, which have, overall, produced a variety of positive effects, such as increases in sensitivity to anticancer drug-induced apoptosis and reductions in proliferation and invasiveness [13]. Based on these observations, the development of clinical inhibitors of HMOX-1 would likely benefit ATL patients by improving the effectiveness of the current chemotherapeutic regimens used to target the malignant cells.

In addition to possibly impeding anticancer drug-effects, an HBZ-mediated increase in HMOX-1 abundance in infected cells may counteract cytotoxic effects caused by other HTLV-1-encoded proteins. The viral protein, Tax, is essential for HTLV-1 replication, as it activates transcription from the 5’ LTR of the provirus [19] and stimulates mitotic expansion of infected cells [93]. This latter function occurs through the dysregulation of a variety of cellular pathways by Tax. While the apparent goal of this process is to promote cell proliferation, it also culminates in the accumulation of ROS/RNS [23, 24]. This effect of Tax has been attributed to inhibition of stress granule formation and constitutive activation of NF-κB signaling, which activates iNOS expression [25, 26]. Paradoxically, ROS generated through the effort to drive mitotic expansion of infected cells can trigger apoptosis or cellular senescence [41, 94]. In connection with these outcomes and our current results, HBZ has been shown to offset Tax-mediated cellular senescence [41, 42]. While attributed to a reduction in NF-κB signaling, this effect may also involve increased expression of HMOX-1 and other antioxidant proteins by HBZ that serve to detoxify Tax-mediated ROS/RNS. Consistent with this premise, HTLV-1-infected T-cell lines with Tax expression (MT-2 and ATL-2s) exhibited low GSH:GSSG ratios, indicative of oxidative stress. In contrast, TL-Om1 cells, which lack Tax expression due to transcriptional repression of the 5’ LTR promoter, displayed a GSH:GSSG ratio consistent with low oxidative stress. More importantly, knocking down HBZ expression in MT-2 cells resulted in a significant decline in oxidative stress, supporting that HBZ plays an important role in ameliorating the cell’s oxidative state.

The HTLV-1-encoded protein, p13, also stimulates ROS production. p13 functions by localizing to the inner mitochondrial membrane where it induces K^+^ influx, leading to mitochondrial swelling and depolarization, and, in turn, increased ROS production. Interestingly, transformed T-cells appear to be more sensitive to the cytotoxic effects caused by these events than primary T-cells, which has led to the proposal that p13 serves to eliminate HTLV-1-infectected T-cells that become transformed as a means of supporting long-term viral persistence in the host. In the context of this model, it is possible that HBZ usurps the role of p13, thereby reinforcing the survival of virally-infected cells that have undergone transformation.

Similar to our findings, another HTLV-1-encoded protein, p30, was recently reported to suppress the cytotoxic effects of ROS [95]. This function originates from the ability of p30 to upregulate TP53-induced glycolysis and apoptosis regulator (TIGAR)[96]). TIGAR is a fructose-2,6-bisphosphatase that supports metabolism through the pentose phosphate pathway, leading to the production NADPH, which can be used to regenerate GSH from GSSG [97]. Interestingly, like HMOX-1, TIGAR is implicated in cancer cell-survival and proliferation [97]. It is important to note that in this previous study, HBZ, in addition to Tax, was implicated in the accumulation of ROS. This discrepancy with our study may be due to the different methods used to measure oxidative stress. While Hutchison *et al*. used the JC-1 dye, which measures mitochondrial membrane potential [95], we used mass spectrometry to quantify GSH:GSSG ratios. Despite the difference between studies, it is interesting that HTLV-1 appears to have adapted separate mechanisms to combat the cytotoxic effects of ROS. It is possible that these mechanisms evolved explicitly to support viral replication through Tax-driven mitotic expansion. However, they may also produce an unintended effect of supporting survival of infected cells that have undergone transformation.

## Materials and methods

### Plasmids and antibodies

The following mammalian expression plasmids have been described: pcDNA-HBZ Sp1-Myc-His (aa 1–206)[34], pcDNA-HBZ-ΔAD-Myc-His (aa 77–206)[50], pcDNA-HBZ-ΔbZIP-Myc-His (aa 1–130) and pcDNA-HBZ-ΔATG [51], pcDNA-HBZ-(LXXAA)_2_-Myc-His [47], pcDNA-HBZ-MutZIP-Myc-His and pSG-HBZ-Myc [44], pCMV-HBZ-FLAG [40], and pSG-Tax-His [98]. Empty vector plasmids pCMV-3Tag-8 and pSG5 were purchased from Agilent Technologies and pcDNA3.1(+)/ Myc-His A was purchased from Invitrogen. The HBZ-MutZIP-Myc sequence from pcDNA-HBZ-MutZIP-Myc-His was inserted into the EcoRI site of pSG5 to generate pSG-HBZ-MutZIP-Myc. The small Maf sequences from pDNR-Dual vectors (DNASU plasmid repository: HSCD00002293, HSCD00005183 and HSCD00004984)[99] were inserted between the BamHI and HindIII sites in pCMV-3Tag-8 to generate pCMV-MafK-FLAG and pCMV-MafG-FLAG. The sequence corresponding to amino acids 401–606 of Nrf2 from pcDNA3-Myc3-Nrf2 (a gift from Yue Xiong; Addgene plasmid #21555)[100] was inserted between the BamHI and XbaI sites in pcDNA3.1(+)/ Myc-His A to generate the Nrf2 dominant negative expression plasmid, pcDNA-Nrf2-DN-Myc-His. The 4xT-MARE sequence (GeneArt Gene Synthesis, Invitrogen) 5’-CTCGAGTCGAGCTCGGAATTGCTGACTCAGCATTACTCTCGTCGAGCTCGGAATTGCTGACTCAGCATTACTCTCGTCGAGCTCGGAATTGCTGACTCAGCATTACTCTCGTCGAGCTCGGAATTGCTGACTCAGCATTACTCTCGGATCCAAGCTT-3’ was inserted between the XhoI and HindIII sites in pGL4.26 (Promega) to generate pGL-4xT-MARE-Luc.

The bacterial expression plasmids pGEX-HBZ and pGEX-KIX have been described [47]. pGEX-4T-2, pRSET A, and pET3A were purchased from GE Healthcare, Thermo Fisher Scientific, and Novagen, respectively. The sequence corresponding to amino acids 1–57 of HBZ from pcDNA-HBZ-Myc-His was inserted between the BamHI and EcoRI sites in pGEX-4T-2 to generate pGEX-HBZ-AD. The small Maf sequences from the pDNR-Dual vectors (above) were inserted between the BamHI and HindIII sites in pRSET A to generate pRSET A-MafF-His and pRSET A-MafG-His. The sequence corresponding to amino acids 120–206 from pcDNA-HBZ-Myc-His was inserted into the BamHI site in pET3A to generate T7-HBZ-bZIP. Newly constructed plasmids were sequenced.

The following antibodies were used: anti-GST Tag (G7781), anti-Actin clone C4 (MAB1501), anti-FLAG M2 (F3165) and anti-Myc clone 4A6 (05–724) were purchased from Millipore-Sigma; anti-His H-15 (sc-803), anti-p300 N15 (sc-584), anti-CBP A22 (sc-369), anti-Nrf2 C20 (sc-722), anti-Bach1 F9 (sc-271211) and anti-HMOX1 A-3 (sc-136960) were purchased from Santa Cruz Biotechnology; anti-VDAC D73D12 (#12454), anti-MEK1/2 L38C12 (#4694) and anti-HMOX1 (#70081) were purchased from Cell Signaling Technology; anti-MafG (ab154318 and ab86524) and anti-6x His (ab9108) were purchased from Abcam; hybridoma anti-MafG were purchased from DSHB (Cat# PCRP-MAFG-1H7, RRID:AB_2618829); and anti-SP1 (21962-1-AP) was purchased from Proteintech. Anti-HBZ serum was a gift from Dr. Mesnard [33].

### Cells and cell culture

HEK 293T/17 (ATCC, ATCC CRL-11268) were cultured in DMEM supplemented with 10% Fetalplex animal serum complex (Gemini Bio-Products), 2 mM L-glutamine, 100 U/ml penicillin, and 50 μg/mL streptomycin. Clonal HeLa cell lines derived from HeLa-S3 (a gift from Dr. Nyborg) expressing HBZ-Myc-His, HBZ-ΔbZIP-Myc-His, HBZ-MutZIP-Myc-His, HBZ-(LXXAA)_2_-Myc-His, and HBZ-ΔATG were cultured in supplemented DMEM maintained under selection with 0.5 mg/mL geneticin (Thermo Fisher Scientific)[44, 68]. Jurkat and CEM cells (a gift from Dr. Nyborg), MT-2 cells (obtained from the NIH AIDS Research Program, #237) and ATL-2s cells (a gift from Dr. Matsuoka) were maintained in supplemented IMDM. TL-Om1 cells (a gift from Dr. Matsuoka) were maintained in supplemented RPMI. MT-2 cells stably expressing an shRNA that targets HBZ-SP1 (V4)(a gift from Dr. Green) were maintained under selection with 1 mg/mL geneticin [38]. MT-2 cells stably expressing an shRNA against GFP (MISSION pLKO.1-puro eGFP shRNA, Thermo Fisher Scientific SHC005), were established and maintained under selection in 1.5 μg/mL puromycin.

### Ethics statement

Blood samples from symptomatic and asymptomatic HTLV-1-infected donors were obtained from the CHU of Martinique and isolated as previously described [58]. Clinical sample collections for research purposes are stored at the Center of Biological Resources of Martinique (CeRBiM). We received approval from the CeRBiM Review Board to use the samples. HTLV-1 AC and patients suffering from TSP/HAM or ATL were recruited according to World Health Organization (WHO) criteria. AC had no neurologic or haematological symptoms. According to the French Bioethics laws, the collection of samples from AC, TSP/HAM and ATL patients has been declared to the French Ministry of Research. Because the protocol is non-interventional, no informed consent was required, as stated by the French Public Health code and, therefore, the study was conducted anonymously.

### RNA extraction, cDNA synthesis, and quantitative real-time PCR

Cells were equalized, cultured overnight, and RNA was extracted using TRIzol Reagent (Invitrogen) according to the manufacturer’s instructions. For assays using the Nrf2-DN mutant, 1 x 10^7^ HeLa cells stably expressing HBZ-Myc-His cells were electroporated with 3.6 μg pMACS CD4 and 16.4 μg pcDNA or 16.4 μg pcDNA-Nrf2-DN-Myc, and positively transfected cells were enriched using the MACSelect System (Miltenyi Biotec) prior to RNA extraction as described [44]. cDNA was synthesized with random hexamers or oligod(T) primers using the RevertAid cDNA synthesis kit (Thermo Fisher Scientific). Quantitative real-time PCR (qRT-PCR) amplification of cDNA was performed as described [44]. For analysis of diluted cDNA, standard curves were generated for each primer pair using serial dilutions of an appropriate experimental sample. PCR efficiencies from all plates and primer pairs ranged from 89.1% to 134.4%, with correlation coefficients >0.95. Undiluted cDNA was used for analysis of *HMOX1* mRNA in T-cell lines. Primer sequences are as follows: HMOX-1, 5’-TGATAGAAGAGGCCAAGACTGCGT-3’ and 5’-TCGCCACCAGAAAGCTGAGTGTAA-3’; FTH1, 5’-CGCCTCCTACGTTTACCTGT-3’ and 5’-AGCATGTTCCCTCTCCTCAT-3’; SQSRM1, 5’-TTCTTTTCCCTCCGTGCTC-3’ and 5’-GGATCCGAGTGTGAATTTCC-3’; TNFRSF1A, 5’-ACGAGTGTGTCTCCTGTAGTAGTA-3’ and 5’- AACCAATGAAGAGGAGGGATAAA-3’; PIM1, 5’-TTCTTCAGGCAGAGGGTCTCTTCA-3’ and 5’-TGTGGAGGTGGATCTCAGCAGTTT-3’; Nrf2, 5’-CCAGCACATCCAGTCAGAAA-3’ and 5’-GACTGAAACGTAGCCGAAGAA-3’; UBE2D2 (housekeeping gene), 5’-TGCCTGAGATTGCTCGGATCTACA-3’ and 5’-ACTTCTGAGTCCATTCCCGAGCTA-3’. For assays using patient-derived cells, sample processing and qRT-PCR were performed as described [101]. PBMCs were depleted of the CD8^+^-T cells to prevent CD8^+^ T cell-mediated killing of the HTLV-1-infected cells in the specimens. Cells were cultured for 5 days prior to being analyzed.

### Quantification of biliverdin levels

HMOX-1 enzymatic activity of was determined as described, but with modifications to accommodate the use of cultured cells [102]. Cells (2 x 10^6^) were treated with 200 μM hydrogen peroxide in low serum media (0.5% Fetalplex) and for 4h to stimulate HMOX-1 activity and then harvested by centrifugation at 4°C, washed with cold PBS, and suspended in 200 μL homogenization buffer (20 mM Tris-HCl [pH 7.5], 250 mM sucrose, 1 mM EDTA) supplemented with protease inhibitor cocktail (Millipore-Sigma, P8340). Cells were homogenized using a Dounce tissue grinder, and lysates containing 200 μg of protein were adjusted to 20 mM Tris-HCl [pH 7.5], 250 mM sucrose, 12.5 μM hemin chloride (Millipore-Sigma, 3741), 1 mM NADPH (Millipore-Sigma, 481973), and 0.025 U bilirubin oxidase (Millipore-Sigma, B0390) in equal volumes. Reactions were incubated at 37°C for 30 minutes, halted by adding an equal volume of 0.1% formic acid in methanol, and supplemented with 0.1 ng/μL Biliverdin d_4_ (an internal standard; Millipore-Sigma, 795089). Samples were then centrifuged at 15,000 x g at 4°C for 20 minutes, and clarified supernatants were analyzed by liquid chromatography mass spectrometry (LC-MS) using an Agilent 1200 series high performance liquid chromatograph connected to an Agilent 6220 time-of-flight (TOF) mass spectrometer. Chromatographic separation was performed using an Agilent Zorbax Eclipse Plus C18 column (3.5 μm, 2.1 × 150 mm) held at 35°C. Mobile phase A consisted of water with 1% formic acid whereas mobile phase B consisted of acetonitrile with 1% formic acid. Flow rate was set to 0.25 mL/min. Initial solvent composition was 10% B which was held for 1 minute, ramped to 55% B over 1 minute, ramped to 90% B over the next 2 minutes, ramped to 100% B in 2 minutes, and held at 100% B for the next 5 minutes resulting in a total analysis time of 11 minutes. The TOF was operated in positive mode and biliverdin along with biliverdin-d4 were quantified using the [M+H]+ ion. Extracted [M+H]+ ion chromatograms were integrated to give biliverdin peak areas which were then normalized by the peak area of biliverdin-d4. Biliverdin/biliverdin-d4 ratios were compared between groups to determine relative biliverdin concentrations.

### Fractionation of cellular proteins

Whole cell extracts were prepared as described [68] using RIPA buffer (50 mM Tris [pH 8.0], 1% Triton X-100, 100 mM NaCl, 1 mM MgCl_2_, 400 nM TSA, 2 μg/mL leupeptin, 5 μg/mL aprotinin, 1 mM phenylmethylsulfonyl fluoride [PMSF], and 1 mM benzamidine). Isolation of membrane fractions was performed as described [57]. Nuclear and cytoplasmic fractions were prepared as described with slight modification [103]. Briefly, 1.5 x 10^6^ PBS-washed cells were suspended in hypotonic buffer (20mM HEPES pH 7.9, 20% [vol/vol] glycerol, 10 mM NaCl, 1.5 mM MgCl_2_, 0.2 mM EDTA, 1 mM DTT, 0.1% NP40, 2 μg/mL leupeptin, 5 μg/mL aprotinin, 1 mM PMSF, 1 mM benzamidine) and ice-chilled for 10 minutes. Samples were then centrifuged at 0.6 x g and 4°C for 5 minutes. Supernatants (cytoplasmic fraction) were collected, and nuclei were lysed by adding 50 μL of RIPA buffer, vortexing and ice-chilling samples for 15 minutes. Lysates were centrifuged at 16,000 x g and 4°C for 15 minutes, and supernatants (nuclear fractions) were collected. Protein concentrations were determined by Bradford assays (Bio-Rad).

### Co-immunoprecipitation (Co-IP) and western blot assays

Co-IP assays were performed as described [68]. For assays with HEK 293T cells, 2 x 10^6^ cells were transfected using TurboFect (Thermo Fisher Scientific) according to the manufacturer’s instructions (plasmid amounts stated in the figure legend). Whole cell extracts were prepared from cells 24 to 48 hours after transfection, and 300 μg of extract was combined with anti-FLAG M2 magnetic beads (Millipore-Sigma, M8823) or protein G agarose beads (Millipore-Sigma, P7700) pre-bound with 2 μg of anti-Myc antibody. For assays with T-cells, 2 mg of whole cell extract were combined with protein G agarose beads prebound with an anti-MafG hybridoma supernatant (DHSB) or normal mouse serum (Jackson ImmunoResearch laboratories, Inc.). SDS-PAGE, Western blot analysis and nitrocellulose membrane imaging were performed as previously described [68].

### Purification of recombinant proteins

pGEX-based plasmids were transformed into *E*. *coli* BL21 codon plus (DE3) (Stratagene). pRSET-A- and pET3A-based plasmids were transformed into *E*. *coli* BL21 (DE3)/pLysS (Stratagene). GST- and 6xHis-tagged proteins were expressed and purified as previously described [48]. T7-HBZ-bZIP was purified using the T7-Tag Affinity Purification kit (Millipore-Sigma, 69025) according to the manufacturer’s instructions. All purified proteins were dialyzed against 0.1M HM (50 mM HEPES [pH 7.9], 100 mM KCL, 12.5 mM MgCl_2_, 1 mM EDTA, 20% [vol/vol] glycerol, 0.025% [vol/vol] Tween 20, 1 mM dithiothreitol [DTT]), aliquoted and stored at -80°C.

### GST pulldown assays

GST pulldown assays were performed as described [58] with the following modifications: glutathione-agarose beads were equilibrated in RIPA buffer containing 1 mM DTT and incubated with 50 pmol of GST-KIX at 4°C for 1 h. Beads were then washed twice with RIPA buffer and combined with whole-cell extract prepared in RIPA buffer. Binding reactions were mixed at 4°C overnight, and beads were subsequently washed four times with RIPA buffer and re-suspended in SDS sample dye. Eluted proteins were analyzed by Western blot.

### Electrophoretic mobility shift assays (EMSAs)

EMSAs using recombinant purified proteins were performed as described with slight modification [48]. Proteins (combined at amounts stated in the figure legends) were incubated for one hour at 20°C and then supplemented with 2 to 10 fmol of ^32^P-end-labeled double-stranded DNA probe, 100 ng of poly(dA)·poly(dT), 1 μg of BSA, and 1 mM DTT in 0.5x TM (100 mM Tris-HCl [pH 7.5], 20 mM MgSO_4_). Reactions were then incubated for one hour at 20°C. Protein/DNA complexes were resolved on 5% non-denaturing polyacrylamide gels at 200 volts at room temperature. Probe sequences are indicated in the figures.

### *In vitro* immobilized DNA-binding assays

Transfection of cells, preparation of nuclear extracts and immobilized DNA-binding assays were performed as described with the following three modifications [68]. First, HEK293T cells (8 × 10^6^ cells) were transfected with 25 μg pcDNA-HBZ-Myc-His and/or 25 μg pCMV-MafG-FLAG (brought to 50 μg of plasmid with the empty vector). Second, for each reaction, 7 pmol of biotinylated double-stranded DNA oligonucleotide was bound to the streptavidin beads (the DNA sequence is shown in the figure). Third, bead-bound protein complexes were washed twice with ITB (20 mM HEPES [pH 7.9], 0.2 mM EDTA, 100 mM KCl, 6.25 mM MgCl_2_, 10 mM ZnSO_4_, 20% [vol/vol] glycerol, 0.01% Triton X-100, 5% BSA, 0.2 mM PMSF, 1 mM benzamidine, 10 μg/mL aprotinin, 10 μg/mL leupeptin, 1 mM DTT), three times with ITB containing 600 mM KCl, and once with PBS.

### Chromatin immunoprecipitation (ChIP) assays

ChIP assays were performed using the Zymo Spin ChIP Kit (Zymo Research) according to the manufacturer’s instructions, but with the following modifications: chromatin was prepared from ~1 x 10^7^ cells, and 150 μg to 200 μg of crosslinked, sonicated chromatin was immunoprecipitated with 5 μg of antibody or normal rabbit serum (negative control, Jackson ImmunoResearch laboratories, Inc.) overnight at 4°C. Crosslinked chromatin was sonicated using a Misonix Sonicator 4000 (20 sec on, 30 sec off for 5 to 15 min depending on the cell line, amplitude 60). Purified ChIP DNA was analyzed by real-time PCR as described [104]. Primer sequences are as follows: *HMOX1* Distal, 5’-CCCTGCTGAGTAATCCTTTCC -3’ and 5’-CTGAGTCACGGTCTAGAGATTTG-3’; *HMOX1* Proximal, 5’-CATTTCTGCTGCGTCATGTTT-3’ and 5’-GTAGGCAGGAGGAAGTGAAAC-3’; *HMOX1* Gene, 5’-CGCCTTCATGATGAGCATAAC-3’ and 5’-GTTATGCTGTACCTCCTCCTC-3’. Standard curves were generated for primer sets using 5-fold serial dilutions of each input DNA from the ChIP procedure and were included on each experimental plate. PCR efficiencies ranged from 95–156%, with correlation coefficients >0.90. Enrichment values were quantified relative to the input as described [105]. Results were derived from using the Gene site in pcDNA cells as a normalization factor, which involved comparing ChIP samples that used the same antibody. Specifically, for a given ChIP sample, results were calculated by dividing the enrichment values obtained for that ChIP sample by the enrichment value for the Gene site in pcDNA cells, which set “pcDNA Gene” to 1.

### Luciferase assays

TurboFect (Thermo Fisher Scientific) was used to transfect 4 x 10^6^ Jurkat cells with 100 ng of pGL4.26 or pGL-4xT-MARE-Luc and 250 ng of the expression vector(s) indicated in the figure legend (the total plasmid quantity was brought to 1 μg with the empty expression vector). For results shown in Fig 7B, cells were co-transfected with 100, 250 and 500 ng of pSG-HBZ-Myc. For results shown in Fig 7F, cells were co-transfected with 162.5, 325 and 650 ng of pcDNA-Nrf2-DN-Myc-His. Cells were processed 24 hours post-transfection using the Luciferase Assay System (Promega) according to the manufacturer’s instructions, and luminescence was measured using a Glomax 20/20 Luminometer. For each expression vector/pair of expression vectors, luminescence from cells co-transfected with pGL4.26 (background) was subtracted from luminescence from cells co-transfected with pGL-4xT-MARE-Luc.

### Quantification of oxidized and reduced glutathione to evaluate oxidative stress

Samples were processed as described with minor variations [106]. Cells were collected, washed with PBS, suspended in 50–100 μL of extraction buffer (2% TCA, 1 mM EDTA), ice-chilled for 15 minutes, vortexed for 45 seconds, and ice-chilled for an additional 15 minutes. Protein concentrations of the cell extracts were determined by Bradford protein assay (Bio-Rad) and equalized to 2 mg/mL by diluting samples with extraction buffer. Samples were centrifuged at 4000 x g at 4°C for 10 minutes, and supernatants were analyzed by LC-MS (the same instruments as above). Standards of reduced L-glutathione (GSH)(Millipore-Sigma, G4251) and oxidized L-glutathione (GSSG) (Millipore-Sigma, G4376) reconstituted in 50 μL of extraction buffer were used for each experiment. Separation was achieved using an Agilent Zorbax Eclipse Plus C18 column 3.5 μm, 2.1 × 150 mm) held at 35°C. Mobile phase A consisted of water with 1% formic acid whereas mobile phase B consisted of acetonitrile with 1% formic acid. Flow rate was held at 0.1 mL/min throughout analysis. Initial mobile phase composition began at 20% B which was ramped up to 100% B over the next 3 minutes and held constant at 100%B for the following 4 minutes resulting in a total analysis time of 7 minutes. The TOF was operated in positive mode. Reduced and oxidized glutathione were identified and quantified based on the [M+H] and [M+2H] ions respectively. Sample concentrations of GSH and GSSG were determined based on the GSH and GSSG standards.

### Cell viability assays

HeLa clones were plated in 96 well plates at 2.5 x 10^4^ per well and allowed to adhere overnight. Culture media were then replaced with low serum media (0.5% serum) containing 40 μM hemin chloride (Millipore-Sigma 3741) or dimethyl sulfoxide (DMSO) (Millipore-Sigma D2650) and cultured for 24 hours. Cell viability was then evaluated using the MTT Cell Growth Assay kit (Millipore-Sigma) according to the manufacturer’s instructions. For T-cell lines, viability was assessed using alamarBlue (Bio-Rad, BUF012) according to the manufacturer’s instructions. Cells were plated in 96 well dark plates at 2.5 x 10^4^ cells per well in low-serum medium prior to treatment with the indicated concentrations of hemin or DMSO and cultured for 24 hours. Chemical inhibition of HMOX-1 activity was achieved using 1-[[2-[2-(4-Bromophenyl)ethyl]-1,3-dioxolan-2-yl]methyl]-1H-imidazole hydrochloride (OB-24, Tocris). Cells were simultaneously treated with 10 μM OB-24, or DMSO, and 20 μM hemin chloride, or DMSO, for 24 hours. Fluorescence was detected using a fluorescent microplate reader (FL600, Bio-Tek). Data are presented in function of vehicle-treated cells for which viability was set to 100%.

### *In silico* analysis

Microarray data sets used in this study are available at NCBI Gene Expression Omnibus (GEO): accession numbers GSE94409 [59] and GSE55851 [60, 61]. For the latter, samples were grouped based on patient disease classification and surface expression of CADM1 and CD7 as described [62, 63]. For each sample, probes corresponding to the *HMOX1* transcript were identified and GEO2R was used to obtain expression values. ChIP-Seq data sets GSE31477 [45] were analyzed using UCSC Genome Browser [107, 108]. Data were visualized using the Human Feb. 2009 (GRCh37/hg19) assembly and samples included GSM935290 (Stanford_ChipSeq_HeLa-S3_MafK_(ab50322)_IgG-rab). Data from GEO accession number GSE94732 [59] were visualized using the Human Mar. 2006 (NCBI36/hg18) assembly. Alignments between these assemblies were performed manually using NCBI Genome Data Viewer and NCBI BLAST.

## Supporting information

S1 Fig Levels of *hbz* and *HMOX1* mRNA following shRNA- and CRISPR-targeting of HBZ. **(A)** shRNA targeting HBZ reduces its expression. qRT-PCR was used to quantify relative HBZ splice 1 (HBZ-SP1) transcript levels in MT-2 cells stably transfected with a vector expressing an shRNA that targets HBZ (shHBZ) and in MT-2 cells transfected with a vector expressing an shRNA that targets GFP (shGFP). Data are an average of three independent experiments and were normalized to shGFP samples (set to 1). Error bars represent SEM. **(B)** CRISPR/Cas9-mediated loss of HBZ expression correlates with a reduction in *HMOX1* transcript levels. The graph was generated from published microarray data [59] and shows the percent reduction in *HMOX1* transcript levels 7 and 8 days after inducing knockdown of HBZ in the ATL cell line, ST1. Data were obtained using GEO2R to analyze the GSM2474937 and GSM2474938 samples with calculations based on averaged values from the nine array features probing for different regions of the *HMOX1* transcript.
(TIF)

S2 Fig Proviral loads from asymptomatic, TSP and ATL patient samples. **(A)** Proviral loads (PVL) of PBMC samples used in Fig 2D. qRT-PCR was used to quantify proviral DNA copy numbers in CD8^+^ T-cell-depleted PBMCs isolated from asymptomatic HTLV-1 carriers (AC), TSP/HAM (TSP) patients and acute ATL (ATL) patients as described [101]. **(B)** In each sample set, proviral loads and *HMOX1* mRNA did not show a significant correlation. Proviral loads and *HMOX1* mRNA were compared by Pearson correlation coefficient for each sample set from Fig 2D.
(TIF)

S3 Fig Nrf2 and Bach1 levels in cytoplasmic and nuclear fractions from HeLa clones stably expressing HBZ or carrying the empty expression vector (pcDNA). **(A-B)** Graphs show levels of nuclear Nrf2 and Bach1 protein normalized to the cytoplasmic levels of each protein (set to 1). **(C-D)** Graphs show percentages of cytoplasmic and nuclear Nrf2 and Bach1 from the total Nrf2 and Bach1 detected. Data for all graphs are an average of three independent experiments. Protein levels were quantified using ImageQuant TL software.
(TIF)

S4 Fig Alignment of large and small Maf protein sequences. Protein alignments were performed with the NCBI Constraint-based Multiple Alignment Tool (COBALT). Basic region and zipper regions are denoted. Highlighted sequences were identified in the preliminary proteomic screen for HBZ-binding partners. Amino acids that are conserved among all seven of the compared protein sequences are denoted by asterisks (*).
(TIF)

S5 Fig HBZ interacts with the small Mafs to form a DNA-bound complex at MAREs. **(A)** *In vitro* GST pulldown assays were performed by pre-binding 50 pmol of recombinant GST-fusion proteins to glutathione-conjugated agarose, then incubated with 30 pmol of purified recombinant MafF-His (lane 1). Bound protein was eluted (lanes 2–4) and analyzed by Western blot with the indicated antibodies. **(B)** Purified recombinant GST-HBZ (8 pmol) and MafG-His (4 pmol) were incubated with immobilized oligonucleotide probes (MARE, MARE MT), or with streptavidin beads alone. DNA-bound proteins were eluted and analyzed by Western blot using the indicated antibodies.
(TIF)

S6 Fig The *HMOX1* distal enhancer contains three MARE sequences that also lie within the peak of HBZ-enrichment in ChIP assays. **(A)** Sequences of the HMOX-1 Distal and Proximal MafK-binding regions, as well as a downstream region used as a ChIP control. The bolded sequences correspond to the three MAREs in the distal peak region (Distal 1–3) and the single MARE in the proximal peak region. PCR primer annealing sites used for ChIP assays are underlined. **(B)** Peak sequences for MafK-enrichment in HeLa cells and HBZ-enrichment in ATL cells align and contain all three distal AREs. Alignments were performed using EMBOSS Needle Pairwise Sequence Alignment tool (European Bioinformatics Institute).
(TIF)
