# Supplementary figures and images for: HTLV-1 basic leucine zipper factor protects cells from oxidative stress by upregulating expression of Heme Oxygenase I
Source: PLoS Pathog. 2019 Jun 28;15(6):e1007922. doi: 10.1371/journal.ppat.1007922 (PMC6623464; doi:10.1371/journal.ppat.1007922)

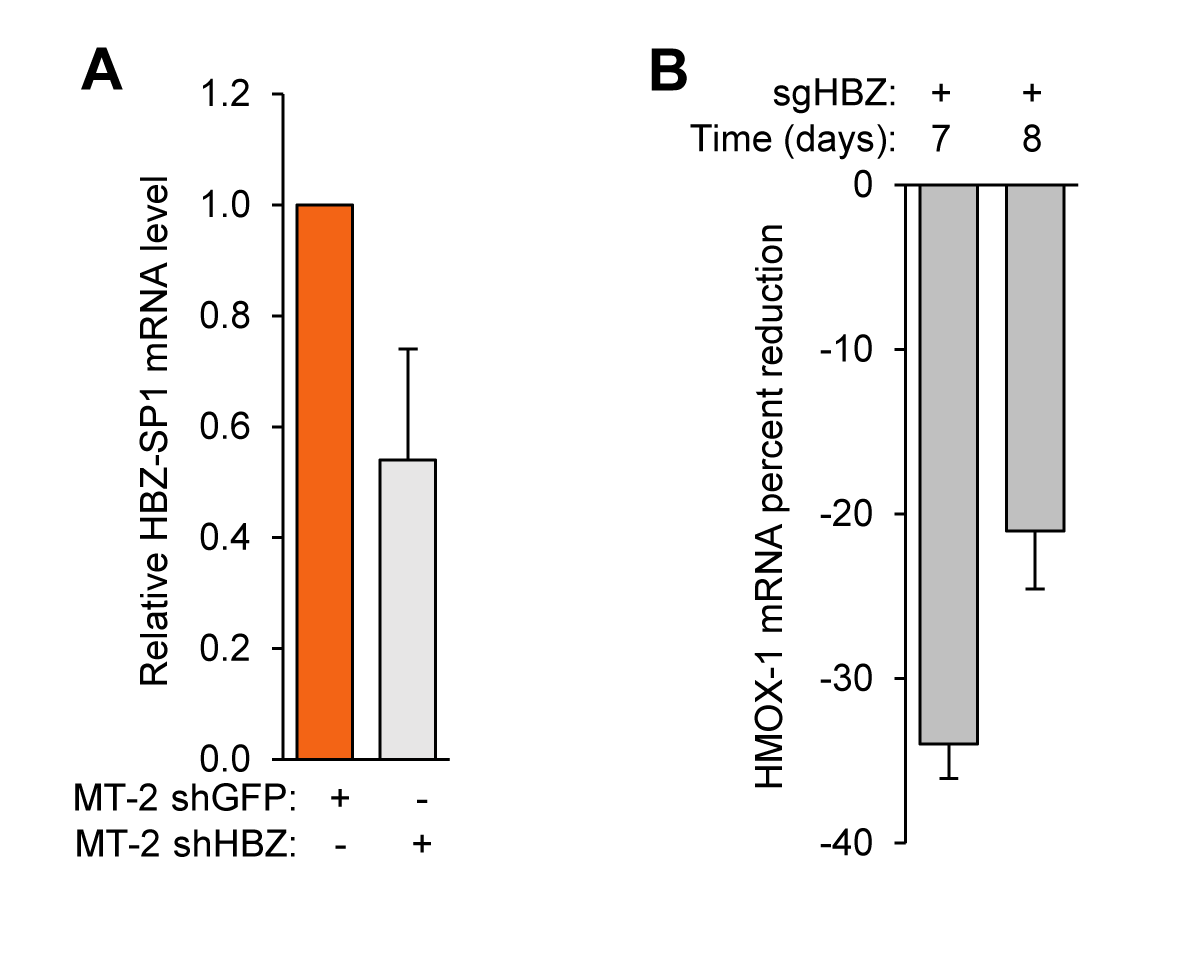

Supplement: S1 Fig — (A) shRNA targeting HBZ reduces its expression. qRT-PCR was used to quantify relative HBZ splice 1 (HBZ-SP1) transcript levels in MT-2 cells stably transfected with a vector expressing an shRNA that targets HBZ (shHBZ) and in MT-2 cells transfected with a vector expressing an shRNA that targets GFP (shGFP). Data are an average of three independent experiments and were normalized to shGFP samples (set to 1). Error bars represent SEM. (B) CRISPR/Cas9-mediated loss of HBZ expression correlates with a reduction in HMOX1 transcript levels. The graph was generated from published microarray data [59] and shows the percent reduction in HMOX1 transcript levels 7 and 8 days after inducing knockdown of HBZ in the ATL cell line, ST1. Data were obtained using GEO2R to analyze the GSM2474937 and GSM2474938 samples with calculations based on averaged values from the nine array features probing for different regions of the HMOX1 transcript. (TIF) [file ppat.1007922.s001.tif]

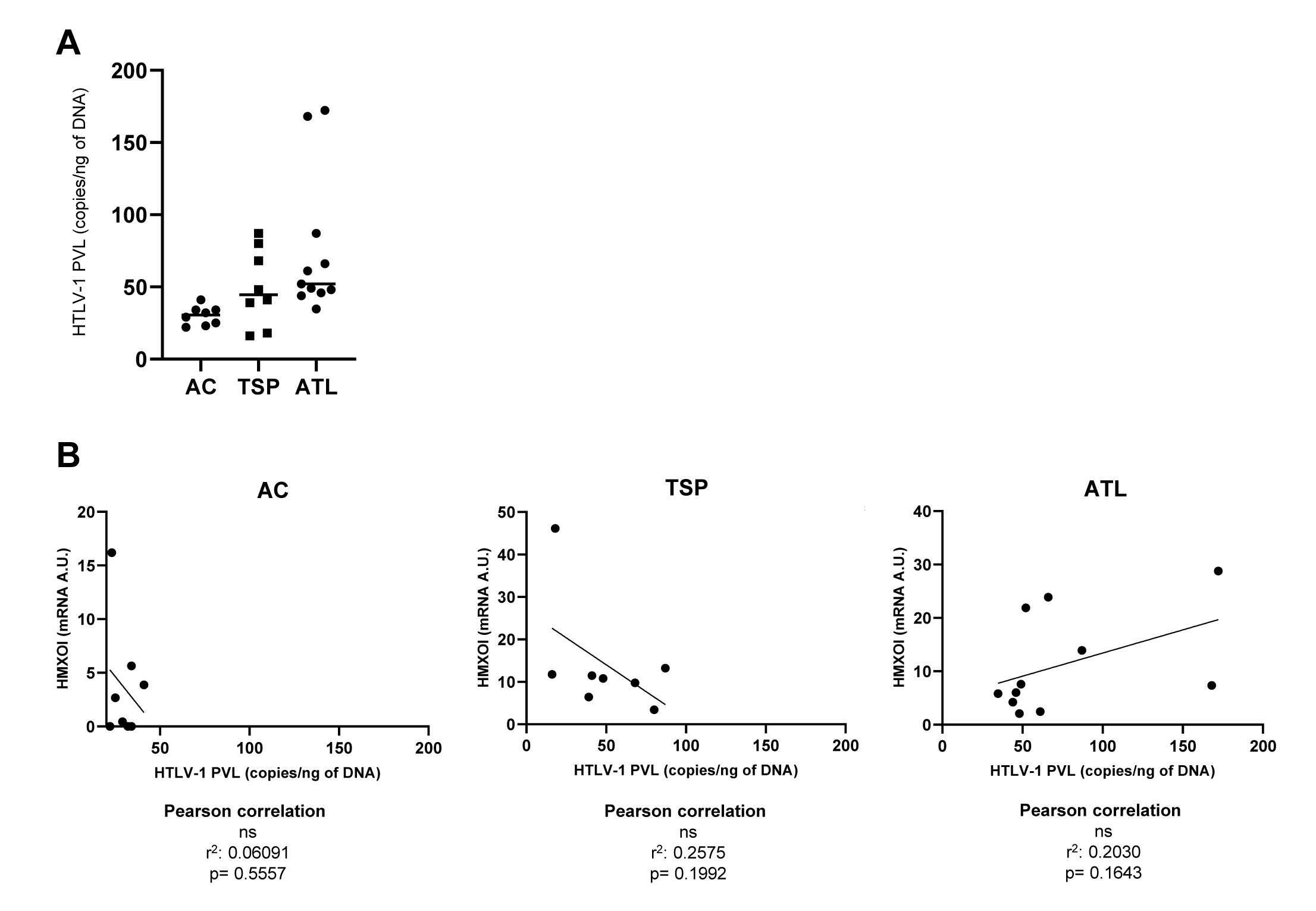

Supplement: S2 Fig — (A) Proviral loads (PVL) of PBMC samples used in Fig 2D. qRT-PCR was used to quantify proviral DNA copy numbers in CD8+ T-cell-depleted PBMCs isolated from asymptomatic HTLV-1 carriers (AC), TSP/HAM (TSP) patients and acute ATL (ATL) patients as described [101]. (B) In each sample set, proviral loads and HMOX1 mRNA did not show a significant correlation. Proviral loads and HMOX1 mRNA were compared by Pearson correlation coefficient for each sample set from Fig 2D. (TIF) [file ppat.1007922.s002.tif]

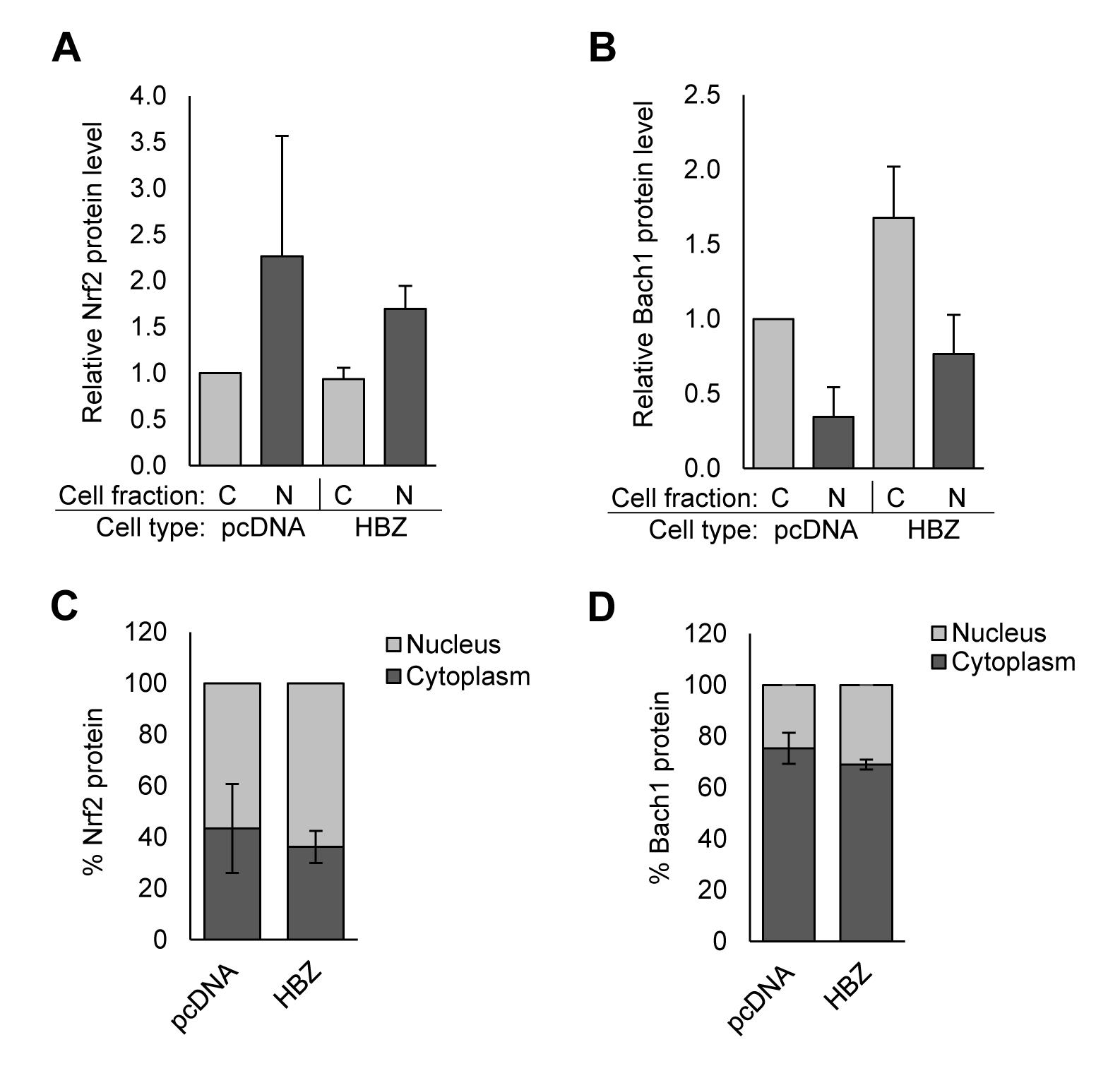

Supplement: S3 Fig — (A-B) Graphs show levels of nuclear Nrf2 and Bach1 protein normalized to the cytoplasmic levels of each protein (set to 1). (C-D) Graphs show percentages of cytoplasmic and nuclear Nrf2 and Bach1 from the total Nrf2 and Bach1 detected. Data for all graphs are an average of three independent experiments. Protein levels were quantified using ImageQuant TL software. (TIF) [file ppat.1007922.s003.tif]

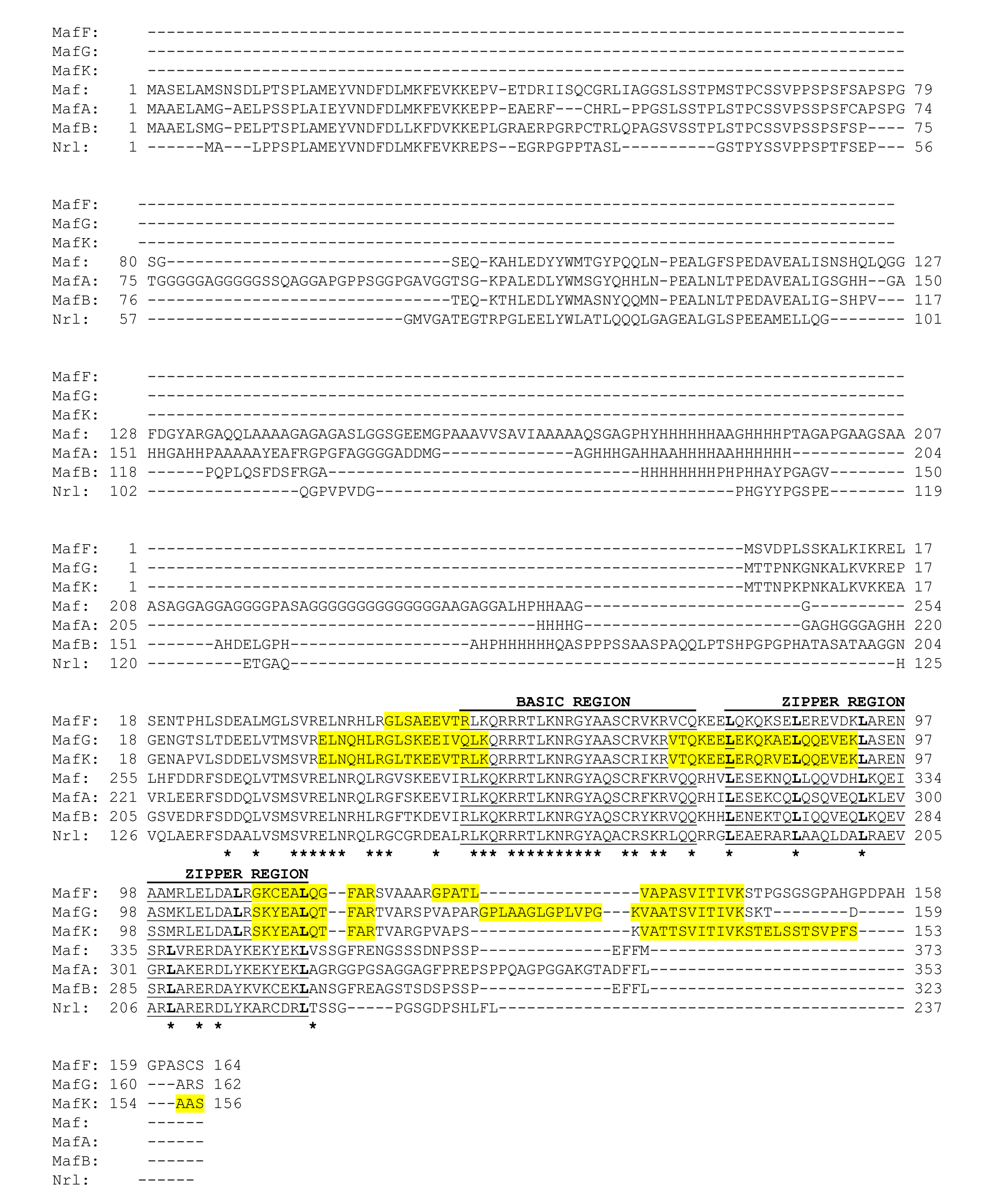

Supplement: S4 Fig — Protein alignments were performed with the NCBI Constraint-based Multiple Alignment Tool (COBALT). Basic region and zipper regions are denoted. Highlighted sequences were identified in the preliminary proteomic screen for HBZ-binding partners. Amino acids that are conserved among all seven of the compared protein sequences are denoted by asterisks (*). (TIF) [file ppat.1007922.s004.tif]

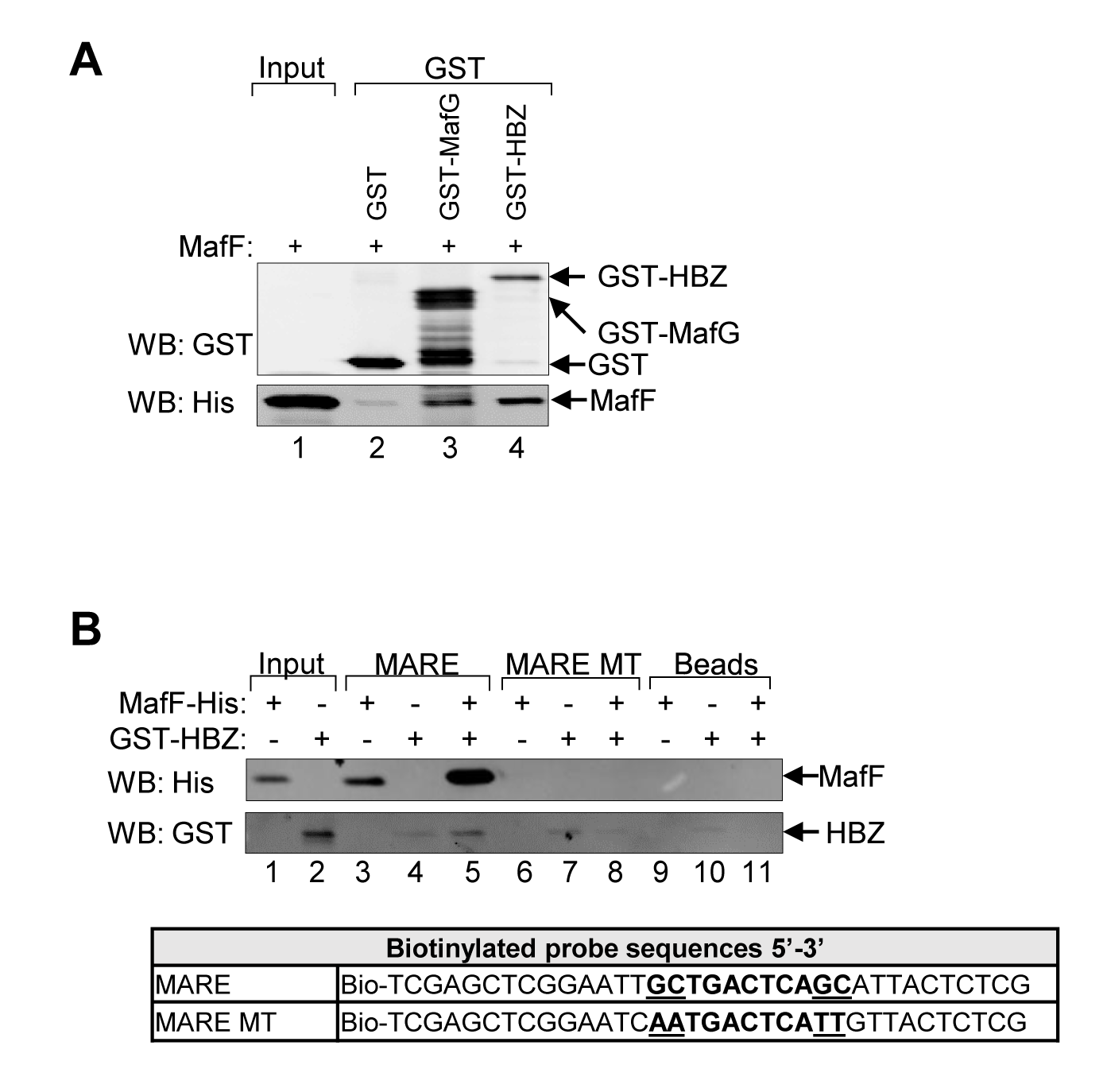

Supplement: S5 Fig — (A) In vitro GST pulldown assays were performed by pre-binding 50 pmol of recombinant GST-fusion proteins to glutathione-conjugated agarose, then incubated with 30 pmol of purified recombinant MafF-His (lane 1). Bound protein was eluted (lanes 2–4) and analyzed by Western blot with the indicated antibodies. (B) Purified recombinant GST-HBZ (8 pmol) and MafG-His (4 pmol) were incubated with immobilized oligonucleotide probes (MARE, MARE MT), or with streptavidin beads alone. DNA-bound proteins were eluted and analyzed by Western blot using the indicated antibodies. (TIF) [file ppat.1007922.s005.tif]

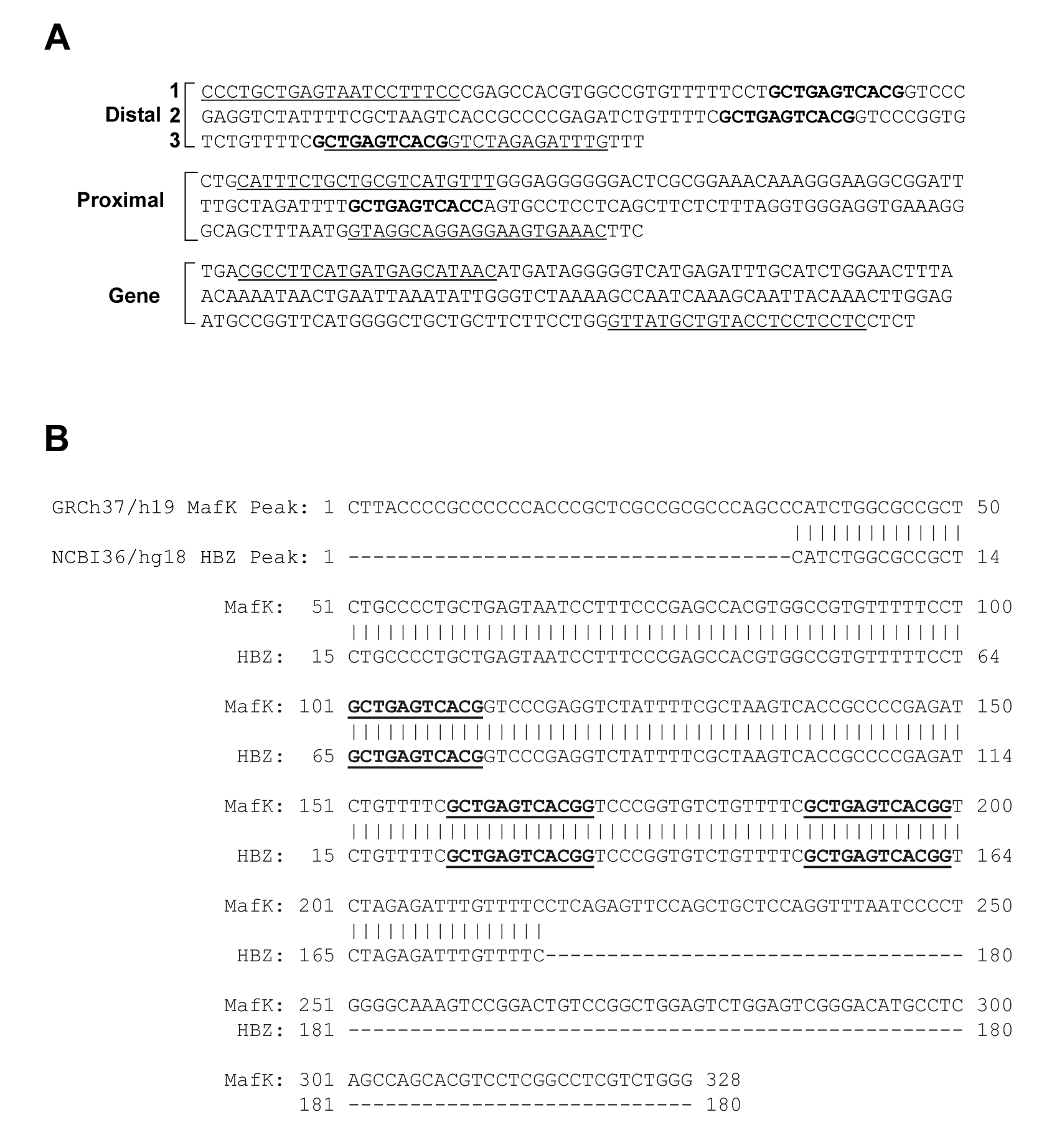

Supplement: S6 Fig — (A) Sequences of the HMOX-1 Distal and Proximal MafK-binding regions, as well as a downstream region used as a ChIP control. The bolded sequences correspond to the three MAREs in the distal peak region (Distal 1–3) and the single MARE in the proximal peak region. PCR primer annealing sites used for ChIP assays are underlined. (B) Peak sequences for MafK-enrichment in HeLa cells and HBZ-enrichment in ATL cells align and contain all three distal AREs. Alignments were performed using EMBOSS Needle Pairwise Sequence Alignment tool (European Bioinformatics Institute). (TIF) [file ppat.1007922.s006.tif]
